# Mental health and wellbeing interventions for uniformed service personnel: a mixed methods systematic review

**DOI:** 10.1186/s12916-026-04811-1

**Published:** 2026-04-17

**Authors:** Jessica Eaton, Holly Ellard, Anita Freeman, Anna M. Anderson, Heather Chesters, Owen J. Arthurs, Alan Simpson

**Affiliations:** 1 Radiology Department, Great Ormond Street Hospital, London, UK; 2 UCL GOS Institute of Child Health, London, UK; 3 NIHR Great Ormond Street Biomedical Research Centre, London, UK; 4 Florence Nightingale Faculty of Nursing, Midwifery and Palliative Care and Health Services and Population Research Department, Centre for Mental Health Nursing Research, King’s College London, London, UK; 5 Psychology and Mental Health Services (PAMHS), Great Ormond Street Hospital, London, UK; 6 Leeds Institute of Rheumatic and Musculoskeletal Medicine, University of Leeds, Leeds, UK; 7 NIHR HealthTech Research Centre in Accelerated Surgical Care, Leeds, UK

**Keywords:** Mental health and wellbeing interventions, Public sector workers, Occupational challenge

## Abstract

**Background:**

Uniformed service personnel are routinely exposed to occupational trauma in their roles which contribute to elevated rates of mental health conditions. A wide range of mental health and wellbeing interventions may help uniformed service personnel manage their psychological responses to challenging incidents at work. Previous reviews of those interventions have focused on single groups of professionals or intervention types, limiting cross-sector insights. This review uses a mixed methods approach to synthesise evidence associated with mental health and wellbeing interventions across uniformed services.

**Methods:**

The protocol was registered with PROSPERO (CRD42024605877). A mixed methods systematic review was conducted using the Joanna Briggs Institute (JBI) convergent segregated approach. Searches were conducted in Medline, CINAHL, Web of Science and PsycINFO (Dec 2024). Screening and quality appraisal (Mixed Methods Appraisal Tool) were performed independently by two reviewers. Due to heterogeneity, findings were narratively synthesised. Quantitative and qualitative results were integrated following the JBI approach.

**Results:**

Eighty-six primary studies from 22 countries were included, covering a range of professions and intervention types. Synthesis of quantitative data from 82 studies showed that mindfulness-based training, resilience coaching, cognitive behavioural therapies and supported lifestyle activities demonstrated reduced symptoms of PTSD, anxiety and depression. However, interventions such as Critical Incident Stress Debriefing (CISD) and peer support yielded mixed results. Qualitative data from 9 studies were grouped into 5 categories: enhanced emotional insight, improved interpersonal relationships, perceived effectiveness, barriers to engagement and delivery challenges. Participants valued interventions that fostered self-awareness and support but cited stigma, guilt and logistical constraints as significant barriers. Integration of quantitative and qualitative findings revealed alignment in outcomes for several interventions, while highlighting evidence gaps, such as the lack of qualitative data for many interventions and limited exploration of cultural and organisational barriers.

**Conclusions:**

Mental health interventions for uniformed service personnel show promise but are influenced by delivery format, occupational culture and contextual factors. This review underscores the need for delivering interventions informed by ecological models, standardised outcome sets and deeper qualitative exploration into stigma and engagement barriers.

**Supplementary Information:**

The online version contains supplementary material available at 10.1186/s12916-026-04811-1.

## Background

Uniformed service personnel, such as healthcare workers, police officers and military workers, face unique challenges in their everyday working lives, due to the ‘high stakes’ nature of their duties and the cultural and organisational frameworks that shape their service delivery [1]. Compared to the general workforce, they are often exposed to unexpected distressing events and unpredictable environments [2]. This can lead to occupational trauma, particularly when coping mechanisms are overwhelmed [3]. These experiences, ranging from natural disasters to violent incidents, graphic accidents or personal attacks, contribute to higher rates of mental health disorders among uniformed service personnel compared to the general population [4]. In turn, ensuring good mental health among uniformed service personnel is essential for operational effectiveness, reducing errors and ensuring safe service delivery [5, 6].

However, stigma and organisational culture often hinder those seeking help, leading to underreported and untreated conditions such as anxiety, depression and burnout [6]. These issues can impair performance, increase absence and strain team dynamics [7–9]. Furthermore, the economic burden of poor mental health is significant, costing billions in lost productivity and turnover [10]; for example, in 2024, nearly 68,000 sick days were taken by London police officers for mental health reasons [11] and on average, every NHS nurse took a full week off work in a year due to stress, anxiety or depression [12].

Several previous systematic reviews have explored whether wellbeing interventions are successful, across multiple public facing professions, including healthcare staff, military personnel, police officers, prison staff, firefighters, rescue workers, transportation officers and retail workers. However, most reviews have focused on a single occupation, such as mental health nursing [13], healthcare workers [14] and first responders [15], limiting cross-sector insights. No review has synthesised findings across the full spectrum of uniformed services, despite shared challenges such as work overload [16], time pressures [17], shift patterns [18] and violence from service users [19].

While existing reviews have predominantly relied on meta-analyses of randomised control trials, this approach often overlooks the valuable insights offered by qualitative and mixed methods research [20]. Including these types of studies is essential for capturing contextual and experiential dimensions of mental health and wellbeing interventions.

A mixed method, cross-disciplinary synthesis is needed to enable shared learning and adaptation of effective interventions across sectors as well as supporting a more integrated understanding of trauma-related mental health challenges across public sector services.

This review aims to answer:

1. What is the evidence for successful mental health and wellbeing interventions for uniformed service personnel who may have experienced occupational trauma?
2. How do uniformed service personnel perceive and experience those mental health and wellbeing interventions?

## Methods

This review followed the Joanna Briggs Institute (JBI) methodology for mixed methods systematic reviews, using a convergent segregated approach. The protocol was registered with PROSPERO (CRD42024605877). The review is reported according to PRISMA guidance [21] (Additional file 1: Table 1).

**Table 1:** Categorisation of interventions

Category	Studies	
**Post-event organisation interventions** *Debriefing, workshops based on acceptance and commitment theory and stress management*	• Adler et al., 2009 • Carlier et al., 2000 • Korpela and Nordquist, 2024	• Leonard and Alison, 1999 • Blevins et al., 2011 • Wu et al., 2012
**Organisational training and support** Mindfulness training, resilience training, Trauma Risk Management, stress training, cognitive behavioural training, moral distress training, meditation training, peer support, supportive leadership training, relaxation skills	• Abbasalizadeh et al., 2024 • Arnetz et al., 2009 • Chitra and Karunanidhi, 2021 • Christopher et al., 2018 • Eddy et al., 2021 • Frappell-Cooke et al., 2010 • Garner, 2008 • Gerdes et al., 2022 • Giaume et al., 2024 • Gon et al., 2023 • Grupe et al., 2021 • Janes et al., 2022 • Jones et al., 2021 • Joyce et al., 2019 • Khatib et al., 2022 • Krick and Felfe, 2020 • Krick and Felfe, 2024 • Leggett et al., 2013 • Lynch et al., 2018 • Mackintosh et al., 2017 • Maloney et al., 2024 • Márquez et al., 2021 • McCall, 2023 • Meland et al., 2015	• Millegan et al., 2021 • Mohr et al., 2024 • Morland et al., 2016 • Navarrete et al., 2022 • Nwokeoma et al., 2019 • Onyishi et al., 2021 • Peng et al., 2024 • Price et al., 2022 • Ramey et al., 2016 • Ranta, 2012 • Reingold, 2015 • Rice et al., 2024 • Rios and Hervas Torres, 2024 • Romosiou et al., 2019 • Said et al., 2022 • Stetz et al., 2011 • Turan and Canbulat, 2023 • Villaruz Fisak et al., 2020 • Wang et al., 2024 • Watson and Andrews, 2018 • Wild et al., 2020
**Psychological therapies** Exposure therapy, cognitive behavioural therapy, motivational enhancement therapy, acceptance and commitment therapy	• Alghamdi et al., 2015 • Brown et al., 2019 • Foa et al., 2018 • Foa et al., 2022 • Maguire et al., 2024 • McLean, Cook, et al., 2024 • Peterson et al., 2023	• Niemeyer et al., 2020 • Rajeswari et al., 2020 • Sloan et al., 2022 • Walker et al., 2024 • Young-McCaughan et al., 2022 • Zarvijani et al., 2021
**Supported lifestyle activity** Integrated lifestyle therapy, rest and recuperation, microbreaks, physical activity	• Chu et al., 2022 • Jones et al., 2013 • Mainsbridge et al., 2020 • Markwell et al., 2016	• McKeon et al., 2023 • Otis et al., 2024 • Rosenbaum et al., 2022 • Walter et al., 2023 • Walter et al., 2019
**Psychoeducation** Psychoeducation sessions delivered in VR format, group sessions and digitally	• Stelnicki et al., 2021 • Van Der Meer et al., 2020	• Wesemann et al., 2016 • Pallavicini et al., 2022
**Integrative treatment programmes** Residential clinics	• Judkins and Bradley, 2017 • Kline et al., 2024	• Smeeding et al., 2010
**Alternative or complimentary therapies** Yoga or dog training	• Nassif et al., 2023 • Scotland-Coogan et al., 2020	• Stoller et al., 2012
**Nontherapeutic** Zentangle, archaeological dig and music	• Hsu et al., 2021 • Nimenko and Simpson, 2014	• Narayanan et al., 2024

*Q1. What is the evidence for successful mental health and wellbeing interventions for uniformed service personnel who may have experienced occupational trauma?*

### Population

The term ‘uniformed services’ broadly refers to government-funded personnel distinguished by their uniforms; these roles, for example, in healthcare, law enforcement and firefighting, are essential to public safety, national security and societal welfare [22]. The term lacks a universal definition, with occupational classifications varying internationally [23].

For this review, the UK government definition [24] was used to define uniformed service personnel occupations. This included the following professions: armed forces, law enforcement, emergency services, healthcare and national support services, e.g. Royal National Lifeboat Institution. Participants were included in this review irrespective of a formal diagnosis of a mental health disorder.

### Occupational trauma

For the purposes of this review, ‘occupational trauma’ refers to exposure to potentially traumatic events encountered in the course of professional duties, such as critical incidents, life-threatening situations or repeated exposure to distressing events. This differs from general work-related stress, which may arise from organisational pressures or workload demands, and from common mental health problems such as anxiety or burnout that can occur in any workplace. Uniformed service personnel often experience both occupational trauma and high stress environments, which interact to shape mental health outcomes. Although occupational trauma is central for many uniformed personnel, this review intentionally included both trauma specific outcomes, e.g. post-traumatic stress disorder (PTSD) and broader mental health and wellbeing outcomes (e.g. anxiety, stress and burnout). This reflects the interconnected nature of trauma exposure and general occupational stressors in uniformed services and ensures the synthesis captures the full range of psychological impacts relevant to this population.

### Eligibility criteria

To account for the diverse ways in which this topic may have been investigated according to profession, intervention and methodology, all primary study types were included. For the purpose of this review, interventions for mental health and wellbeing were defined as ‘….any type of local or outside support that aims to protect and promote psychosocial well-being and/or prevent or treat mental health conditions’ [25].

#### Inclusion criteria

- Intervention focused on improving mental health and wellbeing in relation to occupational exposure to distressing incidents
- Primary studies only

#### Exclusion criteria

- Not available in English language
- Pharmaceutical interventions
- Studies focusing on COVID-19
- Systematic reviews or meta-analyses
- Studies with participants who were students or non-uniformed workers. Studies presenting mixed uniformed and non-uniformed occupations

Pharmaceutical interventions were excluded as this review was conceptually and methodologically focused on non-pharmacological and psychosocial approaches to mental health and wellbeing. These interventions differ substantially in delivery, mechanisms and evaluation from pharmacological treatments, and therefore pharmaceutical interventions were beyond the intended scope of this synthesis. Studies related to COVID-19 were excluded due to the unique nature of the pandemic which may not reflect typical and current occupational challenges. Studies involving students or trainees were excluded a priori, regardless of their level of responsibility or exposure to occupational trauma. This decision was made to maintain conceptual consistency, as the review aimed to synthesis evidence relevant to fully qualified uniformed personnel. Students typically operate under different supervision, training requirements and organisational contexts, which may influence both their experiences and effectiveness of interventions.

### Information sources

The following databases were searched from inception: Medline (Ovid), CINAHL (EBSCOhost), Web of Science Core Collection and PsycINFO (Ovid). The searches were conducted in December 2024.

### Search strategy

An experienced member of library staff from University College London was consulted when building the search. The searches included subject headings and text words related to uniformed services (population), common mental health and wellbeing support terms (intervention) and common mental health problems (condition).

*Population terms* included a range of uniformed and emergency service roles as per the UK government definition [24] such as aid worker, army officer, coastguard, diver, firefighter, scenes of crime personnel, navy, paramedic, police officer, prison officer, Royal Air Force, Royal Marines, soldier, armed forces, healthcare professionals, allied health professionals, radiographers and surgeons. These were combined with occupational descriptors such as work, occupation, job, first responder, NHS and employment.

*Intervention terms* focused on pre- and post-exposure services and included peer support, debriefing, psychological interventions, psychological first aid and stress management.

*Condition terms* targeted mental health outcomes and included psychosocial, emotional wellbeing, psychological health, psychological distress, stress, mental health, anxiety, burnout, post-traumatic stress disorder (PTSD), traumatisation, adverse events, compassion fatigue, occupational stress, posttraumatic stress injury (PTSI) and critical incident.

Boolean operators (AND/OR) were used to combine terms across the three domains. Truncation symbols (e.g. *) were applied where appropriate to capture variations of root terms. The full search strategy was adapted for each database (Additional file 2: Table 2).

Search terms were broad in scope to capture the range and diversity of populations, interventions and conditions that may be relevant to the review question. Outcomes were not specified due to the diverse indicators of mental health and wellbeing and ways in which these are described. No further limits were applied to the search strategies.

### Study selection

**Fig. 1:**
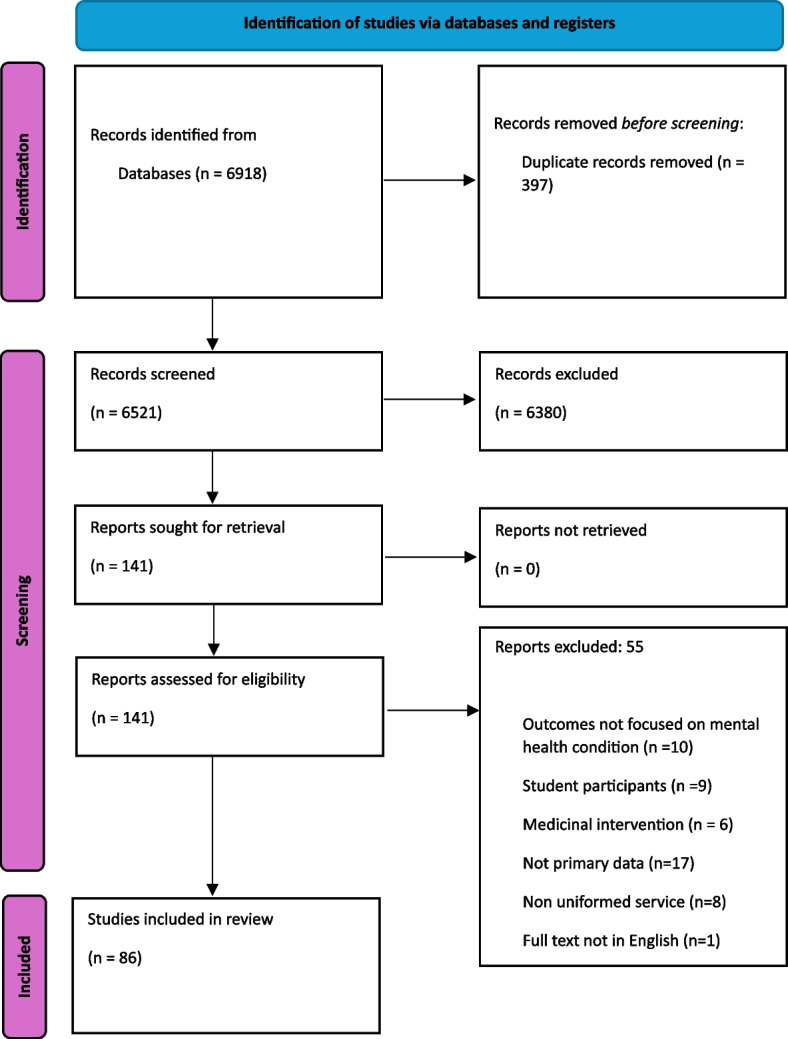
PRISMA 2020 flow chart [29]

All titles and abstracts of potentially eligible studies were independently screened by two authors (JE, HE) against the criteria stated above using Rayyan software. Full-text articles were obtained for all included studies and further reviewed for eligibility by two authors (JE, HE). Discrepancies that could not be resolved were adjudicated by a third reviewer (OA). The study selection process illustrated in Fig. 1.

### Data extraction

#### Data extraction and synthesis

The first author (JE) extracted data from all included studies using Microsoft Excel. Two separate forms were used for quantitative and qualitative data.

Quantitative data.

Quantitative data extracted included country, study type, population/sample size, intervention, description of intervention, follow-up period, data collection tool, outcomes and main findings. Each of the interventions were evaluated by an experienced clinical psychologist (AF) and grouped into intervention categories.

This review intentionally adopted a broad definition of mental health and wellbeing interventions, consistent with JBI guidance, and included multiple professional groups without restricting outcomes. This breadth was a deliberate methodological choice to enable an exploratory, cross-sectoral synthesis of evidence across uniformed services, acknowledging that it would result in a heterogeneous data set. A narrative synthesis was planned from the outset.

Qualitative data.

Qualitative data extracted included country, population, intervention, description of intervention, data collection, outcomes and main findings.

Qualitative data were pooled to determine the experiences and perceptions of uniformed service personnel using mental health interventions.

When following the JBI guidelines, meta-aggregation is usually followed. However, the diversity in theoretical frameworks and research contexts among the qualitative studies in this review made it difficult to meaningfully combine their findings into a single, coherent summary. Therefore, a narrative summary was deemed appropriate.

### Quality assessment

The Mixed Methods Appraisal Tool (MMAT) [26] was used, with no modifications, to appraise all included studies. The MMAT evaluates 25 criteria on five categories of studies (qualitative, RCT, non-randomised studies, quantitative and mixed methods studies). To ensure reliability while balancing feasibility, one reviewer (JE) conducted MMAT quality appraisal, with a second reviewer (HE) independently verifying a random 25% of appraisals. Disagreements were resolved through discussions.

No overall quality ratings were generated, in line with MMAT guidance and PRISMA guidelines. The MMAT ratings were used to inform interpretation of findings. Specifically, studies with lower methodological quality were considered when discussing confidence in the evidence base and identifying areas where further research is needed.

### Data integration

This involves juxtaposing the synthesised quantitative and qualitative findings and linking these into an argument to produce an overall ‘configured analysis’ [27].

As suggested by the JBI guidance [28], the following questions guided this process:

- Are the results/findings from each individual synthesis supportive or contradictory?
- Does the qualitative evidence explain why the intervention is or is not effective?
- Does the qualitative evidence help explain differences in the direction and size of effect across the included quantitative studies?
- Which aspects of the quantitative evidence are/are not explored in the qualitative studies?
- Which aspects of the qualitative evidence are/are not tested in the quantitative evidence?

## Results

### Study selection

Electronic database searches identified 6918 records (including duplicates). After screening titles and abstracts, 141 full-text articles were considered for eligibility (see Fig. 1). Six discrepancies were resolved between JE and HE and none required a third review. Ultimately, 86 studies were included in the review (Additional file 3: Table 3).

### Quality assessment

Using the MMAT criteria, 26 studies were rated as low risk, 43 medium and 17 high risks of bias (Additional file 4: Table 4).

A minority of included studies were deemed high quality and most demonstrated methodological limitations that warrant cautious interpretation of their findings.

Studies viewed as high quality demonstrated good research designs and executions, including appropriate sampling techniques, adequate handling of missing data, good reporting of themes inclusive of quotations and clear reporting of blinding procedures in randomised control trials. These studies adhered closely to the MMAT criteria across categories.

Those that demonstrated lower quality included limitations such as high attrition rates, lack of blinding, incomplete outcome data and limited adjustment for confounders in statistical analyses. Studies deemed at the highest risk of bias included inadequate reporting of intervention fidelity, insufficient power and high participant attrition rates.

### Characteristics of included studies

The included studies were from 22 different countries: USA (39), UK (7), Australia (6), Germany (4), China (4), Netherlands (3), Spain (3), India (3), Nigeria (2), Iran (2), Canada (2), Sweden (1), Saudi Arabia (1), France (1), Taiwan (1), Norway (1), New Zealand (1), Italy (1), Palestine (1), Turkey (1), Finland (1) and Ireland (1).

Study types included randomised controlled trials (39), pilot studies (13), non-randomised trials (12), qualitative (4), quasi-experimental (3), experimental (3), programme evaluations (3), observational (4), mixed methods (2), longitudinal (2) and cohort (1). Categorisation of study designs followed the descriptions provided in each paper. Where no explicit description was available, the classification was based on an assessment of the study’s methodology and design characteristics by the lead reviewer. Study populations included nurses, military, firefighters, police, navy, a range of healthcare workers, doctors, paramedics, radiographers, emergency services, crown prosecutors and search and rescue. Several studies included a variety of occupations. The follow-up periods of the interventions for these studies ranged from 1 to 730 days.

### Types of interventions

Interventions were classified by an experienced clinical psychologist (AF) and assigned one of eight categories: post-event organisation interventions (6 studies), organisational training and support for trauma prevention (45 studies), psychological therapies (13 studies), supported lifestyle activities (10 studies), psychoeducation-based interventions (4 studies), integrative treatment programmes (3 studies), alternative or complementary therapies (3 studies) and non-therapeutic interventions (3 studies) (Table 1).

A total of 82 studies reporting quantitative data were included in answering this question. Findings are summarised below by intervention category. Interventions were assessed using a range of outcome measures and tools. For example, six studies that assessed debriefing used 16 different outcomes and tools, reflecting the complexity and variability in evaluating interventions across multiple occupations.

#### Post-event organisation interventions (5 studies)

Evidence on effective post-trauma debriefing was mixed; Battle Mind Debriefing showed modest improvements in PTSD, depression and sleep problems among US army personnel [30], whereas CISD yielded inconsistent outcomes [31, 32]. Trauma-informed models reduced PTSD, anxiety and depression [33].

#### Organisational training and support (42 studies)

Mindfulness-based interventions (13 studies) demonstrated reductions in negative symptoms associated with challenging workloads with large effect sizes in police populations [34, 35]. Resilience training demonstrated benefits for first responders and police officers, including stress resilience [34, 35], though results varied.

Cognitive behavioural interventions, especially Rational Emotive Occupational Health Coaching (REOHC) [36], demonstrated substantial reductions in organisational stress and improvements in life satisfaction [37].

Emotion regulation training and integrative psychoeducational programmes [38] have been shown to lead to sustained improvements in emotional intelligence, stress, empathy, depression and resilience, with some effects persisisting for up to 2 years following the intervention [39]. In some instances, these types of interventions address workload and pacing by enhancing workers psychological capacity to cope with demanding and fast paced environments. Nurses participated in a psychological group intervention, which resulted in significant improvements in job satisfaction indicators, including a measurable reduction in perceived workload (*p* = 0.036) [40]. Similarly, among police officers, a multidimensional psychological intervention produced significant reduction in job stress (*F*[ (1,72) = 8.046, *p* < 0.001), indicating increased perceived control over work demands [41].

Peer support had mixed outcomes. Trauma Risk Management (TRiM) was associated with reduced PTSD symptoms in some studies [42], while others found limited impact on burnout or quality of life [43]. Other outcomes, such as reduce loneliness and improved supervisor support, were noted [44].

Digital interventions, including mobile apps and VR, were effective at reducing anxiety levels, anger and PTSD symptoms [45, 46]. Digitally delivered self-compassion tasks improved emotional wellbeing [47], but there were considerable individual differences in psychophysiological responses.

Culturally adapted psychological first aid (PFA) interventions yielded variable outcomes. While some studies reported modest improvements in self-efficacy and reductions in depression and burnout [48, 49], others demonstrated substantial gains in psychological preparedness [50].

Supportive supervision was shown to be an important organisational factor in two studies. A targeted supportive leadership programme for US army leaders resulted in reduced loneliness at 4-month follow-up (*b* =  − 0.46, *p* < 0.05) and destigmatising behaviour (*b* = 0.09, *p* < 0.01) for employees alongside increased perceived supervisor emotional support (*b* = 0.07, *p* < 0.05) [44].

#### Psychological therapies (13 studies)

Many of the interventions in this group were used for uniformed service personnel identified to have PTSD and used as a treatment option. Exposure-based therapies (e.g. prolonged exposure, narrative exposure therapy) demonstrated PTSD symptom reductions [51]. Internet-based cognitive behavioural therapy demonstrated small, but not statistically significant changes in PTSD and small significant improvements in anxiety [52].

#### Supported lifestyle activity (9 studies)

Physical activity interventions demonstrated improved depression and PTSD [53, 54]. Passive recovery showed limited benefits to users [55]. Integrated programmes combining movement and diet improved psychological distress and quality of life [56].

#### Psychoeducation (4 studies)

Programmes like Before Operational Stress [57] and virtual reality-based tools [58] improved PTSD symptoms, resilience and emotional wellbeing. Technology-based delivery was prominent, with mobile apps and computer-based systems showing cognitive and emotional benefits.

#### Integrative treatment programmes (3 studies)

Residential programmes, such as Freedom Restoration Clinic (FRC) [59] and OASIS [60] for the armed forces, demonstrated mixed findings. While the FRC clinic demonstrated reduced psychological distress, the OASIS programme found that residual symptoms of PTSD and depression were common, even among service members who reported a clinically significant reduction in PTSD symptoms.

#### Alternative or complementary therapies (3 studies)

Mindfulness yoga combinations and sensory-enhanced yoga reduced depression and anxiety [61, 62]. Dog training programmes showed broad symptom reductions in trauma-impacted veterans [63].

#### Nontherapeutic (3 studies)

Creative and decompression activities, including Zentangle art [64] and archaeological digs [65], reduced psychological distress and enhanced wellbeing. Music interventions showed limited impact on participants [66].

#### Summary of Q1 findings

Organisational training, mindfulness and cognitive behavioural approaches showed consistent reductions in PTSD, anxiety and depression. Trauma-informed models outperformed traditional debriefing methods in outcomes such as burnout. Lifestyle activities, psychoeducation and integrative treatments improved emotional wellbeing and resilience. Digital and culturally adapted interventions yielded mixed but promising results. Alternative therapies like yoga and dog training also demonstrated benefits. However, outcome measures varied widely across studies, highlighting the complexity of evaluation intervention effectiveness in diverse occupational contexts.

### Q2. How do uniformed service personnel perceive and experience those mental health and wellbeing interventions?

A total of nine studies were included in the qualitative synthesis. Five studies were also analysed in the quantitative section above due to using multiple approaches in their design.

Qualitative synthesis included papers studying 263 police officers [31, 32, 57, 67], 23 healthcare professionals [68], 48 firefighters [57, 69], 25 paramedics [57], 10 emergency department staff [70], 89 army personnel [45, 71] and 11 crown prosecutors [57].

A range of study designs were used: qualitative, control group design, non-randomised trial mixed methods study, process evaluation and randomised control trial. A range of data collection techniques were used including interviews, qualitative survey and focus groups.

The participants were exposed to interventions including Critical Incident Stress Debriefing (CISD) [31, 32, 69], resilience training [68], mindfulness-based resilience training (MBRT) [67], mantra meditation (MM) [70], web-based prolonged exposure therapy (web-PE) [71], Before Operational Stress programme (BOS) [57] and technology-assisted relaxation training [45].

The findings were narratively summarised in five categories relating to individuals’ perceptions and experiences of mental health and wellbeing interventions in the workplace.

#### Enhanced self-awareness and emotional insight (4 studies)

Participants reported gains in self-awareness and deeper emotional insights following engagement with some interventions. Improved recognition and understanding of emotions for individuals attending the Before Operational Stress (BOS) programme—divided into eight weekly groups for psychoeducation and group processing was noted [57]. Police officers reported being more aware of the present moment and experienced improved physical wellbeing when using a mindfulness-based resilience training intervention [67]. Similarly, emergency service personnel gained greater compassion and acceptance towards their self and others using debriefing [69], and military personnel reported better processing of traumatic memories using web-based prolonged exposure therapy [72].

#### Improved relationships and support (4 studies)

Participants highlighted the positive impact some interventions had on their relationships and support from others. Mindfulness-based resilience training demonstrated improvements in family communication at home and camaraderie with colleagues [67]. Similarly, an increased openness with family and peers, alongside an increased willingness to seek and offer help to others, was also reported using debriefing [69] and mantra meditation [70]. This was reinforced by findings that a psychoeducational intervention improved relationships with family and colleagues and fostered feeling less alone and more supported during troubling times at work [57].

#### Perceived effectiveness (4 studies)

Perceptions of intervention effectiveness were mixed. While some participants reported positive outcomes, others highlighted challenges associated with using the interventions. Increased emotional awareness was reported from CISD [32] and the Before Operational Stress (BOS) psychoeducational intervention found aggravation of symptoms or revived memories of past exposures [57]. Motivation was noted by some participants as a barrier to using a web-based prolonged exposure therapy intervention [72] and reports of dissatisfaction due to poor follow-up from CISD sessions [32].

#### Barriers to engagement and practice (6 studies)

A range of structural, cultural and personal factors hindered individuals from fully engaging with interventions. Demanding work schedules, shift patterns and long intervention times made it difficult for participants to attend or sustain participation in mindfulness-based resilience training [67] and mantra meditation training [70]. These challenges were increased by participants’ additional responsibilities including home and work commitments.

Cultural and organisational barriers, such as working in high-pressure environments, often made individuals feel guilty for taking time to focus on their mental wellbeing. Guilt, combined with a broader cultural stigma around mental health support, discouraged participants from engaging fully in practices like meditation [70]. Similarly, cultural stigma was a barrier to participation [67]. Some participants reported struggling to prioritise their own needs, while others found it difficult to maintain motivation [70, 71].

#### Digital delivery (2 studies)

Despite the potential benefits of digital interventions, studies highlighted technical and structural limitations that hindered user experience. Technical difficulties when using a web-based platform was reported including issues such as freezing, slow performance and login problems with the intervention [72]. Longer sessions and more flexible scheduling options were also desired [45].

#### Summary of Q2 findings

Participants attributed enhanced emotional insight, improved self-awareness and strengthened relationships with family and colleagues to interventions. While some found interventions effective, others experienced temporary emotional distress or dissatisfaction. Poor follow-up was noted, highlighting a potential risk/safety consideration needed for these types of interventions. Barriers to engagement included demanding work schedules, cultural stigma and organisational pressures. Digital interventions showed promise but were hindered by technical issues and inflexible delivery formats. Overall, experiences highlighted the need for tailored, accessible and well-supported mental health strategies in high-pressure occupational settings.

### Integration

The findings from the quantitative and qualitative syntheses were largely supportive of one another. Specific interventions such as mindfulness-based training [33, 70, 71], resilience training [35, 40] and CBT-based coaching [36, 37] yielded statistically significant improvements in PTSD symptoms, depression, anxiety and overall wellbeing. Qualitative findings reinforced these results, with participants reporting enhanced emotional insight, improved relationships and greater self-awareness [57, 67, 69].

However, CISD presented an inconsistency between quantitative and qualitative findings. While some quantitative studies reported increased re-experiencing of traumatic events [31], qualitative data suggested emotional gains, but also dissatisfaction [32, 71] and temporary symptom worsening. Qualitative reports suggested that poor follow-up, emotional distress and lack of organisational support may be reasons for mixed results. Similarly, peer support interventions, like TRiM, which follow similar delivery methods in group settings, showed inconsistent quantitative outcomes [42]; qualitative data suggested that trust, openness and stigma influence their effectiveness [57].

Many interventions did not have a qualitative exploration of participants’ experiences. All studies evaluating integrative residential programmes and alternative therapies included in this review were quantitative. Outcomes such as heart rate variability and blood pressure levels reported in quantitative studies [73, 74] were lacking qualitative narratives, for example, what participants were feeling at the time of increased heart rate. Qualitative data may provide context and explanations for the outcomes. Vice versa, many factors identified through qualitative studies were not explored using quantitative measures. Barriers to engagement, such as cultural stigma, guilt and workload, were absent from quantitative measurements and delivery issues for technically delivered intervention such as technical problems were rarely quantified.

## Discussion

This systematic review explored the impact, lived experience and evidence of success of mental health and wellbeing interventions for uniformed service personnel exposed to occupational trauma. Across the 86 included studies that met our eligibility criteria, most interventions were associated with improvements in PTSD symptoms, anxiety, depression and stress. However, differences in delivery formats, outcome measures, quality of studies and contextual factors complicated generalisability.

### Intervention delivery

Delivery methods varied widely, including group-based, individual, digital and hybrid formats, with different modalities, durations and facilitators. The context in which interventions were offered also differed, some targeted individuals experiencing PTSD symptoms or mental health difficulties, while others were broader, workplace-based preventative approaches. Group-based resilience training showed mixed outcomes, while individual coaching demonstrated more consistent benefits. Digital interventions offered accessibility and reduced stigma but faced engagement and monitoring challenges. Hybrid models, such as FIRECARE [75], demonstrated reduction in burnout for emergency health care professionals.

To understand these varied outcomes, it would be helpful to evaluate intervention delivery within ecological frameworks that consider the relationship between individual, organisational and policy-level factors. These frameworks help evaluate interventions within the broader systems that influence their implementation and effectiveness, e.g. healthcare interventions can be significantly shaped by organisational culture, leadership support and NHS policies, all of which can influence whether and how such interventions are adopted and sustained.

The use of implementation science frameworks, such as The Consolidated Framework for Implementation Research (CFIR) [76], offers a structured approach to explore these dynamics. Applying CFIR, or other applicable frameworks, in future studies could help identify barriers and facilitators to implementation and guide adaptation strategies to enhance fit within specific service contexts. This approach moves beyond assessing effectiveness alone and enables a deeper understanding of how and why interventions succeed or fail in real-world settings.

### Outcomes

We identified multiple different outcomes using diverse tools, even for similar intervention types. This lack of standardisation limited synthesis and comparability. For example, trauma focused debriefing interventions used varied psychological, functional and attitudinal measures, which demonstrated mixed results across our included studies. Mixed results in peer support initiatives further underscored the need for consistent evaluation frameworks. The development of Core Outcome Sets (COS), such as those in psychological therapies or mental health services [77, 78], could improve comparability and policy relevance across occupational contexts.

Although this review focused on the outcomes for uniformed service personnel themselves, an important gap relates to the role of external support networks for those individuals, e.g. families. Despite evidence that family members often absorb secondary impacts of occupational trauma [79], few included studies explicitly examined or reported this, and those that did were not captured in the data extraction process. This limits understanding of how interventions might influence, or be strengthened by, the broader support networks surrounding employees. Future research which includes these perspectives could be a key opportunity to broaden intervention design and ensure support mechanisms reflect the ecological realities of uniformed service work.

### Barriers to engagement and access

Stigma around mental health remained a significant barrier. Uniformed personnel often perceived help seeking as weakness, impacting engagement and wellbeing. The review also highlighted additional organisational and moral pressures that constrained engagement. High-pressure work environments and rigid workload expectations contributed to feelings of guilt when prioritising personal wellbeing. These factors, alongside cultural stigma, shaped participation in interventions such as mindfulness and therapy. Indeed, wider literature has demonstrated prejudice against people who have mental illness, and discrimination against people diagnosed with mental illness [80]. Addressing these barriers requires organisational strategies that normalise mental health support, embed interventions into routine practice and challenge cultural norms that discourage self-care. Future research should include qualitative studies exploring how organisational culture, workload demands and self-sacrifice influence intervention uptake. Such insights could inform implementation strategies that integrate mental health support into daily work life, reduce stigma and enhance acceptability [81].

### Limitations to this systematic review

Our review methods present limitations. The broad definition of ‘uniformed services’ excluded some occupational subgroups, and demographic reporting was often insufficient, restricting insights into diverse experiences. Language restrictions and publication bias may have further skewed findings. Furthermore, findings were not synthesised according to profession type, which may have obscured profession-specific intervention effects.

The search strategy did not include grey literature, or citation searching, which may have resulted in the omission of relevant studies. The search was conducted in December 2024. This reflects a pragmatic decision given the scope and complexity of these mixed methods review. Future reviews should build on this work by incorporating more recent evidence to maintain currency and further strengthen the knowledge base in this area.

Several limitations and gaps were identified across the studies included in this review using the MMAT quality criteria. Many lacked detailed intervention descriptions, hindering categorisation. Short follow-up periods and post-intervention-only evaluations limited an understanding of long-term impacts, despite the ongoing nature of workplace trauma. Moreover, the broader literature has identified that health professionals with personal histories of trauma are at increased risk of experiencing secondary traumatic stress when caring for individuals in distressing circumstances [82], yet this was largely overlooked in the baseline assessments and analyses of most studies in this review. Additionally, the optimal timing for intervention following exposure to traumatic events was not explored, leaving a gap in understanding of when support may be most effective.

While study screening was performed by two researchers, data extraction was conducted by a single reviewer. This may introduce a risk of extraction error, which should be considered when interpreting the results.

## Conclusions

This review highlights both the potential benefits of mental health and wellbeing interventions for uniformed staff in demanding roles, as well as the complexity of implementing such interventions within these services. By adopting a cross-sector, mixed methods approach, this review addresses a key gap in the literature, the absence of integrated evidence spanning multiple uniformed professions and intervention types. Unlike previous reviews that focus narrowly on effectiveness or single occupation groups, this synthesis combines quantitative outcomes with qualitative insights to provide a more comprehensive understanding of what works, for whom and under what conditions. Findings demonstrate that intervention success is shaped not only by delivery methods, but also by organisational culture and contextual factors. Future research should build on this broader perspective by prioritising ecological delivery models, standardised outcome sets and deeper qualitative exploration of barriers such as stigma, workload pressures and cultural norms. This will strengthen the evidence base and support tailored implementation strategies across uniformed services.

## Supplementary Information

Additional file 1: Table 1 PRISMA 2020 checklist.

Additional file 2: Table 2 Search strategy.

Additional file 3: Table 3 Full data extraction data.

Additional file 4: Table 4 Risk of bias assessments.

## Data Availability

Full table of data extraction is available upon request.

## Abbreviations

JBI: Joanna Briggs Institute
CISD: Critical Incident Stress Debriefing
PTSD: Post-traumatic Stress Disorder
PTSI: Posttraumatic Stress Injury
MMAT: Mixed Methods Appraisal Tool
REOHC: Rational Emotive Occupational Health Coaching
TRiM: Trauma Risk Management
PFA: Psychological First Aid
FRC: Freedom Restoration Clinic
BOS: Before Operational Stress Programme
CFIR: The Consolidated Framework for Implementation Research
COS: Core Outcome Sets
