# Supplementary material for: Mental health and wellbeing interventions for uniformed service personnel: a mixed methods systematic review
Source: BMC Med. 2026 Apr 17;24:333. doi: 10.1186/s12916-026-04811-1 (PMC13217766; doi:10.1186/s12916-026-04811-1)
Supplement: Supplementary file 2 — Additional file 2: Table 2 Search strategy. [file 12916_2026_4811_MOESM2_ESM.docx]

1. What interventions are available to address the mental health impacts on emergency and uniform services exposed to occupation stress?

| Population | | | Intervention | And | Condition |
| --- | --- | --- | --- | --- | --- |
| Uniform services | AND | Occupation | Pre- and Post-Exposure Services |  | Post Traumatic Stress injuries |
| Aid worker  Or  Army officer  Or  Coastguard  Or  Diver  Firefighter  Or  Scenes of crime  Or  Navy  Or  Paramedic  Or  Police Officer  Or  Prison Officer  Or  Royal Air Force  Or  Royal Marines  Or  Soldier  Or  Army  Or  Armed Forces  Or  Healthcare  Or  Allied Health Profession*  Or  Radiographer*  Or  Surgeon |  | Work*  Or  Occupation*  Or  Job*  Or  First Responder*  Or  NHS  Or  Employment | Peer Support  Or  Debrief  Or  Psychological intervention*  Or  Psychological First Aid  Or  Stress Management |  | Psychosocial  or  Emotional well-being  or  Wellbeing  or  Psychological health  Or  Psychological distress  Or  Stress  or  Mental Health  Or  Anxiety  Or  Burnout  Or  Post-Traumatic Stress Disorder  Or  PTSD  Or  Traumatization  Adverse Events  Or  Compassion fatigue  Or  Psychological distress  Or  Occupation stress  Or  Posttraumatic stress injury  Or  PTSI  Or  Critical incident |
