# Supplementary material for: Mental health and wellbeing interventions for uniformed service personnel: a mixed methods systematic review
Source: BMC Med. 2026 Apr 17;24:333. doi: 10.1186/s12916-026-04811-1 (PMC13217766; doi:10.1186/s12916-026-04811-1)
Supplement: Supplementary file 3 — Additional file 3: Table 3 Full data extraction data. [file 12916_2026_4811_MOESM3_ESM.docx]

| Author | Country | Study Type | Population | Intervention | Description of Intervention | Intervention Delivery | Follow up period | Theory | Data Collection Tool | Outcomes | Results |
| --- | --- | --- | --- | --- | --- | --- | --- | --- | --- | --- | --- |
| (Abbasalizadeh et al., 2024) | Iran | Randomised controlled trial | 60 ICU nurses | **Resilience Training via mHealth Application**:  **Control Group**: No additional training. | Included educational videos, practical demonstration videos, and voiced PowerPoints covering topics such as resilience, proper breathing techniques, self-esteem, self-compassion, hope, effective communication, and problem-solving skills. | Five months  Initial in-person meeting followed using the app | 5 months | Not reported | Personal information questionnaire  Depression, anxiety and stress scale (DASS-21) | Stress  Anxiety | **Intervention Group**:  Stress 10.77 ± 3.33 to 9.00 ± 1.66, P=0.001*P*=0.001)  Anxiety (from 9.43 ± 3.35 to 7.93 ± 0.98, P<0.001*P*<0.001).  **Control Group**:  Stress levels (from 10.10 ± 2.19 to 10.73 ± 2.15, P=0.002*P*=0.002)  Anxiety levels (from 9.10 ± 1.63 to 10.23 ± 1.65, P<0.0001*P*<0.0001). |
| (Adler et al., 2009) | United States | Randomised Control Trial | 2,297 army personnel | **Battle mind Debriefing**  **Battle mind Training**  Control: Traditional stress management strategies. | **Battle mind Debriefing:** Psychological debriefing adapted for the military context, focusing on transitioning from combat to home.  **Battle mind Training**: Cognitive and skills-based program designed to help soldiers reintegrate post-deployment. | **Debriefing**: Small groups of 20-32 soldiers, median duration of 50 minutes.  **Training**: Small groups of 18-45 soldiers or large groups of 126-225 soldiers, median duration of 39 minutes.  **Stress Education**: Large groups of 51-257 soldiers, duration between 40-50 minutes | 4 months |  | Demographic questionnaire  Subjective units of distress scale (SUDS)  Combat exposure scale  Post traumatic stress disorder checklist (PCL)  Patient health questionnaire for depression (PHQ-D)  Sleep problems scale  Stigma scale  Post intervention evaluation survey | PTSD  Depression  Sleep problems  Stigma associated with seeking mental health care | **PTSD Symptoms**  Battle mind debriefing: 35.76 (SD = 15.31) versus 38.92 (SD = 14.78) (d) of 0.21  Battle Mind Training: Adjusted mean PCL score 36.82 (SD = 14.72) versus 38.92 (SD = 14.78) (d) of 0.14  Depression symptoms  **Battle mind Debriefing**:.82 (SD = 0.67) versus 1.99 (SD = 0.64) (d) of 0.26  **Battle mind Training**: Adjusted mean PHQ-D score was 1.95 (SD = 0.69) versus 1.99 (SD = 0.64) (d) of 0.06  Sleep problems  **Battle mind Debriefing**: Adjusted mean probability 0.26 (SD = 0.65) versus 0.42 (SD = 0.72) (d) of 0.25  **Battle mind Training**: Adjusted mean probability 0.26 (SD = 0.58) versus 0.42 (SD = 0.72) (d) of 0.25  Stigma  **Battle mind Training**: Adjusted mean stigma score 2.79 (SD = 0.88) versus 3.01 (SD = 0.86) (d) of 0.25 |
| (Alghamdi et al., 2015) | Saudi Arabia | Randomised control trial | 34 firefighters | Narrative Exposure Therapy (NET) | Four therapy sessions of 60–90 minutes over three weeks.  Participants created and revised a detailed autobiography of traumatic experiences with a therapist, reliving the experiences to transform emotional memories into coherent narratives. | Delivered by trained researchers | Immediate post treatment, 3 months and 6 months | Not reported | Scale of Posttraumatic Stress Symptoms (SPTSS)  Hospital Anxiety and Depression Scale (HADS)  Adapted Brief COPE scale  Jaber’s Social Support scale | PTSD  Anxiety  Depression symptoms  Coping strategies and perceived social support | **PTSD Symptoms** **NET pre to post-treatment:** Mean difference: **6.65** 95% CI: **(5.23 to 8.05)** t (16) = **10.01**, **p < .001**, Effect size (Hedge's g): **0.75.**  **Between-group (NET vs WLC at post-treatment):** F (1,31) = **102.5**, **p < .001** Effect size (Hedge’s g): **2.05**  **Anxiety (HADS)**  **NET pre to post-treatment:** Mean difference: **2.00.** 95% CI: **(1.27 to 2.72).** t (16) = **3.82**, **p < .01.** Effect size: **0.58**  **treatment):** **Passive Coping Strategies** **NET group pre to post-treatment:** Mean difference: **2.18** 95% CI: **(1.05 to 3.30)** t (16) = **4.74**, **p < 0.001** Effect size: **0.52**  **Between-group at post-treatment:** F (1,31) = **1.29**, **not significant** **Follow-up (3- and 6-Months)** Repeated Measures ANOVA for PTSD:  Time effect: F (2,31) = **2.43**, **p = 0.05.**  No sustained effect at 3- or 6-months |
| (Arnetz et al., 2009) | Sweden | Randomised controlled trial | 18 police officers | Imagery and Skills Training Program  Control Group: Training as usual | Initial psychoeducational session followed by ten weekly, 2-hour small group sessions.  Relaxation techniques, imagery training using verbally presented scripts of various critical incident traumas (CITs), cognitive and behavioural skills training, and group discussions | Small groups of ten or fewer participants.  Facilitated by national special forces senior officers trained by the researchers | 12 months |  | Blood samples  Heart rate  Observations of behaviour by blinded expert  Mood states inventory  Visual analogue scale of perceived stress | Negative mood  Antithrombin levels  Police work performance  Cortisol levels  Stress | **Negative Mood**: Imagery-trained officers reported significantly less negative mood compared to the control group (effect size d=−1.11*d*=−1.11, p=0.03*p*=0.03).  **Heart Rate**: Imagery-trained officers had significantly less heart rate increase during the critical moment (handcuffing) compared to the control group (effect size d=−1.60*d*=−1.60, F=26.82*F*=26.82, p=0.00*p*=0.00).  **Antithrombin Levels**: Imagery-trained officers had significantly greater increases in antithrombin levels compared to the control group (effect size d=1.03*d*=1.03, F=4.82*F*=4.82, p=0.04*p*=0.04).  **Police Work Performance**: Imagery-trained officers performed significantly better than the control group (effect size d=1.26*d*=1.26, p=0.02*p*=0.02).  **Cortisol Levels**: Imagery-trained officers had a smaller decrease in cortisol levels compared to the control group, although this difference was not statistically significant after adjusting for baseline levels (effect size d=0.89*d*=0.89, p=0.43*p*=0.43).  **Self-Reported Stress**: Imagery-trained officers reported less increase in self-reported stress compared to the control group (effect size d=−0.80*d*=−0.80, F=2.65*F*=2.65, p=0.13*p*=0.13) |
| (Blevins et al., 2011) | USA | Quasi experimental | 144 Army personnel | Lifeguard workshop based on Acceptance and Commitment Therapy (ACT) | A 2-hour, interactive ACT-based workshop focused on three skills: awareness, acceptance, and value-based living. Delivered through skits, metaphors, and role-playing to promote resiliency and facilitate reintegration. | Delivered by a team including a nurse, social worker, psychologist, and recreational therapist | 2 month follow up | Not reported | PHQ-9 (Depression)  GAD-7 (Anxiety)  PTSD Checklist – Civilian (PCL-C)  Dyadic Adjustment Scale (DAS)  SF-12 (Global Health)  AUDIT (Substance Use)  Conflict Tactics Scale (CTS) | Depression  Anxiety  PTSD  Relationship satisfaction  Anger  Physical Mental Health  Substance use  Interpersonal conflict | **Depression**  Intervention: **–2.209**, 95% CI: (–3.58, –0.84), **p = 0.002**  Between-group difference: **2.609**, 95% CI: (0.71, 4.51), **p = 0.008**  **Anxiety**  Intervention: **–1.492**, 95% CI: (–2.92, –0.06), **p = 0.044**  Between-group: not statistically significant (**p = 0.136**)  **PTSD**  Intervention: **–4.561**, 95% CI: (–8.05, –1.07), **p = 0.011**  Between-group: marginally non-significant (**p = .098**)  **Relationship Satisfaction**  Intervention: **+2.515**, 95% CI: (1.24, 3.79), **p < 0.001**  Between-group: **–2.621**, 95% CI: (–4.34, –0.90), **p = 0.004**  No significant changes in physical health, interpersonal conflict, anger, or substance use |
| (Brown et al., 2019) | USA | Randomised control trial | 326 army personnel | **Spaced Prolonged Exposure Therapy (S-PE)**  **Massed Prolonged Exposure Therapy (M-PE)**  **Present-Centred Therapy (PCT)** | **Prolonged Exposure (PE):** Involves imaginal and in-vivo exposure to trauma-related cues.  **PCT:** Supportive, non-directive therapy focusing on current stressors rather than trauma. | Delivered by trained clinicians following manualized protocols for each therapy  **(S-PE):** 10 sessions over 8 weeks  **(M-PE):** 10 sessions over 2 weeks  **(PCT):** 10 sessions over 8 weeks | Post-treatment, 2 weeks, 3 months, and 6 months |  | PTSD Symptom Scale – Interview (PSS-I)  Beck Depression Inventory-II (BDI-II)  Posttraumatic Cognitions Inventory (PTCI)  PTSD Checklist (PCL) | PTSD Severity  Trauma related cognitions  Depressive symptoms | **S-PE**  PSS-I score at 6 months:  Rapid responders: M=17.78, symptom exacerbation: M=33.10 (d) = 3.83. Significant differences in PTSD, depression, BDI-II and PTCI across all classes (p<0.001)  M-PE  PSS-I score 6 month follow up:  Rapid responders M=13.74, symptom exacerbation M=27.35, (d) = 2.16  PCT  PSS-I at post treatment:  Steep responders M=11.44, symptom exacerbation M=29.35, p<0.001 |
| (Carlier et al., 2000) | Netherlands | Nonrandomised trial | 243 police officers | Critical Incident Stress Debriefing (CISD) | Included traumatic stress education and followed a seven stage, semi structured procedure. | Delivered by 43 trained police officers and supervised by three police social workers.  Three debriefing sessions held at 24 hours, 1 month, and 4 months. | 6 months | Not reported | Spielberger State Trait Anxiety Inventory (STAI)  Self-Rating Scale for PTSD (SRS-PTSD)  Impact of events Scale (IES)  Peritraumatic Dissociative Experiences Questionnaire (PDEQ-R)  Structured Interview for PTSD (SI-PTSD)  Anxiety Disorders Schedule Revised (ADIS-R) | Psychological morbidity  PTSD symptomatology  Satisfaction with debriefing  Sick leave  Work Resumption | **No significant reduction in psychological morbidity**: Debriefing did not lead to a reduction in psychological morbidity at pre-test, 24 hours, or 6 months post-trauma.  **Increased PTSD symptomatology**: One week post-trauma, debriefed subjects exhibited significantly more PTSD symptomatology compared to non-debriefed subjects (44% vs. 26%, χ² (1) = 10.2, p < .01).  **No significant differences in sick leave or work resumption**: There were no significant differences between the debriefed and non-debriefed groups in terms of sick leave or work resumption rates. |
| (Chitra & Karunanidhi, 2021) | India | Nonrandomised trial | 63 police officers | Resilience training program | Components such as self-awareness, positive attitude, emotional management and interpersonal skills | 20 group sessions each lasting 1.5 hours held thrice a week for 2 months. | 2 months | Protective model of resilience | Occupational stress inventory (OSI)  Connor Davidson Resilience Scale (CD-RISC)  Overall job satisfaction scale modified from Brayfield and Rothes scale  Psychological General Wellbeing Index (PGWBI) | Occupational Stress  Resilience  Job Satisfaction  Psycholoigcal wellbeing | **Occupational Stress**: (pre-assessment: 78.96 ± 28.87, post-assessment: 60.00 ± 25.35, follow-up: 54.69 ± 27.46).  **Resilience**: (pre-assessment: 59.69 ± 11.13, post-assessment: 71.77 ± 12.45, follow-up: 74.58 ± 11.37).  **Job Satisfaction**: (pre-assessment: 32.15 ± 8.69, post-assessment: 42.81 ± 5.02, follow-up: 42.15 ± 5.51).  **Psychological Well-being**: (pre-assessment: 38.19 ± 10.58, post-assessment: 48.88 ± 7.40, follow-up: (51.04 ± 8.12). |
| (Christopher et al., 2018) | USA | Randomised Controlled Trial | 61 police officers | Mindfulness based resilience training (MBRT) | Body scan, sitting and walking meditations, mindful movement and group discussion. Content and language adapted specifically to law enforcement | Eight weekly 2 hour sessions with extended 6 hour session in the seventh week.  Participants given iPad with guided practices to support practice between sessions | 3 months | Mindfulness Based Stress Reduction Framework (MBSR) | PROMIS  Concise Health Risk Tracking Scale (CHRT)  Police Stress questionnaire  Oldenburg Burnout Inventory (OLBI)  Five facet mindfulness questionnaire short form (FFMQ-SF)  Acceptance and Action Questionnaire 2 (AAQ-II)  Self-Compassion Scale Short Form (SCS-SF)  Connor Davidson Resilience Scale (CD-RISC)  Bus Perry Aggression Questionnaire Short Form (BPAQ-SF)  Salivary Cortisol collected at 0,30 and 45 minutes after awakening on 3 consecutive days pre and post training | Psychological health and risk outcomes  Aggression  Stress reactivity | **Psychological Health and Risk**: Significant improvements in burnout (p = 0.006; d = 0.73), organizational stress (p = 0.05; d = 0.52), FFMQ non-reactivity (p = 0.04; d = 0.60), and psychological flexibility (p = 0.006; d = 0.73). Trend-level improvement in sleep disturbance (p = 0.08; d = 0.60). No significant changes in anxiety, depression, suicidal ideation, operational stress, resilience, nonjudging, acting with awareness, and self-compassion.  **Aggression**: Significant reduction in aggression (p = 0.03; d = 0.55). No significant changes in anger.  **Cortisol Awakening Response**: Significant reduction in cortisol increases after awakening on day three post-training (p = 0.02; d = -0.70). |
| (Chu et al., 2022) | Hong Kong | Randomised control trial | 18 police officers | Integrated lifestyle medicine program | Five lifestyle factors: physical activity, diet, mindfulness, sleep and positive psychology. | Delivered by registered dietician, traditional Chinese medicine practitioner, yoga teacher and clinical psychology trainee under supervision  Each session lasted 2.5 hours. | 1 week | Not reported | Patient health questionnaire (PHQ-9)  Depression. Anxiety and Stress Scale-21 (DASS-21)  Insomnia Severity Index (ISI)  Short Form Health Survey (SF-6D)  Sheehan disability scale (SDS)  Health promoting lifestyle profile (HPLP-11)  Credibility Expectancy Questionnaire (CEQ)  Brief Resilience Scale (BRS) | Psychological wellbeing  Quality of life  Treatment credibility  Health behaviours | **PHQ-9**: Significant reduction in depression scores in the intervention group (pre-intervention: M = 11.17, SD = 2.48; post-intervention: M = 7.50, SD = 2.43; t (5) = 2.56, p = 0.05, d = 1.05).  **SF-6D**: Significant reduction in QoL scores in the intervention group (pre-intervention: M = 2.64, SD = 0.45; post-intervention: M = 1.56, SD = 0.69; t (5) = 2.57, p = 0.05, d = 1.05).  **CEQ**: Significant increase in treatment credibility scores in the intervention group (pre-intervention: M = 5.83, SD = 0.35; post-intervention: M = 6.56, SD = 0.54; t (5) = -2.74, p = 0.04, d = 1.13).  **Other Measures**: No statistically significant differences between pre- and post-intervention were observed for DASS-21, ISI, SDS, HPLP-II, and BRS |
| (Villaruz Fisak et al., 2020) | USA | Pilot study | 40 Navy | Buddy care. A peer to peer intervention | 1 hour meetings  follow up meetings determined by the pair | Promoted through monthly trainings, emails and weekly visits to the unit by Buddy care providers. | 3 months and 6 month interventions | Not reported | Response to Stressful experience scale (RSES)  Perception of safety  Horizontal Cohesion  Perceived Stress Scale  Burnout Measure | Resilience  Perceptions of safety  Cohesion  Stress level  Burnout | **Perception of Safety**: Large effect size from baseline to 3 months (0.9), lowered at 6 months (0.14).  **Burnout Measure**: Medium effect size from baseline to 3 months (0.37), lowered at 6 months (0.00).  **Statistical Significance**: No statistically significant differences were found across the time-points. |
| (Foa et al., 2018) | USA | Randomised control trial | 370 military personnel | Prolonged Exposure therapy (PE) | Massed therapy (10 sessions over 2 weeks)  Spaced therapy (10 sessions over 8 weeks)  Present centred therapy (10 sessions over 8 weeks) | Participants randomised to receive mass therapy, space therapy, present centred therapy or minimal contact control (MCC) | Post treatment, 2 week, 12 week and 6 months | Not reported | PTSD symptom scale interview (PSS-I)  PTSD checklist stressor specific (PCL-S)  Veterans RAND 12 item health survey  Adverse events | PTSD symptom severity  Treatment efficacy  Treatment efficiency  Adverse events | **PSS-I**: 2-week follow-up, massed therapy showed a significant decrease in PSS-I scores (mean decrease = 7.13) compared to MCC (mean decrease = 3.43), with a difference in decrease of 3.70 (95% CI, 0.72 to 6.68; P = .02).  **PCL-S**: Massed therapy showed a significant decrease in PCL-S scores (mean decrease = 15.52) compared to MCC (mean decrease = 6.13), with a difference in decrease of 9.38 (95% CI, 4.85 to 13.92; P < .001).  **PTSD Diagnosis**: Massed therapy had a lower rate of PTSD diagnosis (54.6%) compared to MCC (77.1%), with a difference of 22.5% (95% CI, 7.5% to 32.8%; P = .005).  **Massed Therapy vs Spaced Therapy**:  **PSS-I**: 2-week follow-up, massed therapy was noninferior to spaced therapy (difference = 0.79; 1-sided 95% CI, - to 2.29; P = .049). 12-week follow-up, massed therapy was noninferior to spaced therapy (difference = 0.55; 1-sided 95% CI, - to 2.05; P = .03).  **PCL-S**: 2-week follow-up, massed therapy was noninferior to spaced therapy (difference = 0.90; 1-sided 95% CI, - to 3.17; P < .001). 12-week follow-up, massed therapy was noninferior to spaced therapy (difference = 0.55; 1-sided 95% CI, - to 2.82; P < .001).  **PTSD Diagnosis**: 2-week follow-up, massed therapy was noninferior to spaced therapy (difference = 2.7%; 1-sided 95% CI, - to 13.8%; P = .043). 12-week follow-up, massed therapy was noninferior to spaced therapy (difference = 0.5%; 1-sided 95% CI, - to 11.5%; P = .02).  **Spaced Therapy vs PCT**:  **PSS-I**: At posttreatment assessment, there was no significant difference between spaced therapy and PCT (difference = 0.10; 95% CI, -2.48 to 2.27; P = .93).  **PCL-S**: Spaced therapy showed a greater decrease in PCL-S scores (mean decrease = 14.91) compared to PCT (mean decrease = 11.33), with a difference in decrease of 3.58 (95% CI, 1.70 to 5.47; P < .001).  **PTSD Diagnosis**: There was no significant difference in the rate of PTSD diagnosis between spaced therapy and PCT. |
| (Foa et al., 2022) | USA | Randomised control trial | 160 military personnel | Prolonged Exposure (PE) therapy | In vivo exposure (approaching trauma related distressing situations) and imaging exposure (repeated recounting of the most distressing traumatic memory). | Therapy delivered by five licensed, masters level counsellors who completed training and received weekly supervision from PE experts.  Sessions were in person or online.  Study compared 60 min sessions with 20 min imaging exposure to 90 min sessions with 40 min imaginal exposure | 3 months and 6 months post treatment | Emotional processing theory | Clinician administered PTSD scale (CAPS-5)  PTSD checklist (PCL-5) | PTSD symptom severity  Treatment efficacy  Treatment efficiency  Adverse events | **CAPS-5**: Posttreatment: 90-min PE (M = 17.99), 60-min PE (M = 15.43), difference = -2.56, upper limit of 95% CI = 1.29, p = 0.278, BF 01 = 3.39.  3-month posttreatment: 90-min PE (M = 16.07), 60-min PE (M = 15.26), difference = -0.81, upper limit of 95% CI = 3.11, p = 0.736, BF 01 = 5.56.  6-month posttreatment: 90-min PE (M = 16.79), 60-min PE (M = 14.55), difference = -2.24, upper limit of 95% CI = 1.86, p = 0.368, BF 01 = 4.03.  **PCL-5**: Posttreatment: 90-min PE (M = 21.31), 60-min PE (M = 21.89), difference = 0.58, upper limit of 95% CI = 7.37, p = 0.887, BF 01 = 5.81.  3-month posttreatment: 90-min PE (M = 23.43), 60-min PE (M = 21.48), difference = -1.95, upper limit of 95% CI = 5.38, p = 0.665, BF 01 = 5.38.  6-month posttreatment: 90-min PE (M = 23.72), 60-min PE (M = 20.71), difference = -3.01, upper limit of 95% CI = 4.80, p = 0.530, BF 01 = 4.88.  **Adverse Events**: 2.5% in 60-min PE reported AEs (one study-related), 10% in 90-min PE reported AEs (two study-related), and three serious AEs in 90-min PE (two increased suicide risk, one suicide, all unrelated to study participation). |
| (Frappell-Cooke et al., 2010) | United Kingdom | Nonrandomised trial | 56 Royal Marines and 91 army personnel | Trauma Risk Management (TRiM) | Peer-group model of psychological risk assessment. | Psychoeducation briefings and trauma risk assessment interviews | Pre deployment, during deployment, post deployment | Peer group model of psychological risk assessment | General Health Questionnaire (GHQ12)  PTSD Checklist civilian version (PCLC)  Combat experience questionnaire | General mental health  Traumatic stress  Social support | Pre-deployment **General Distress (GHQ12 ≥ 4)**: Coldstream Guards: 21% (18 out of 86). Royal Marines: 8% (7 out of 94)  **Odds Ratio (OR)**: 4.0 (95% CI, 1.54–10.35) for those not previously deployed; 1.5 (95% CI, 0.44–5.02) for those previously deployed.  **Trauma-related Stress (PCL(C) ≥ 50)**: Coldstream Guards: 7% (6 out of 86). Royal Marines: 1% (1 out of 94)  **Odds Ratio (OR)**: 6.0 (95% CI, 1.92–19.0) for those previously deployed; 2.5 (95% CI, 1.05–5.90) for those not previously deployed. During Deployment **General Distress (GHQ12 ≥ 4)**: Coldstream Guards: 18% (9 out of 49) Royal Marines: 22% (12 out of 56)  **Trauma-related Stress (PCL(C) ≥ 50)**: Coldstream Guards: 6% (3 out of 49). Royal Marines: 2% (1 out of 56) Post-deployment **General Distress (GHQ12 ≥ 4)**: Coldstream Guards: 11% (5 out of 46). Royal Marines: 3% (3 out of 91)  **Trauma-related Stress (PCL(C) ≥ 50)**: Coldstream Guards: 2% (1 out of 46). Royal Marines: 1% (1 out of 91)  **Correlation with Trauma-related Stress (PCL(C))**: Royal Marines: r = -0.28 (P < 0.05) during deployment. Coldstream Guards: r = -0.32 (P < 0.05) during deployment |
| (Garner, 2008) | USA | Nonrandomised trial | 63 Police officers | Criticism management and stress inoculation training program | Included three steps conceptualisation, skills acquisition and application | 16 hours of classroom instructions  Booster sessions delivered via video at 1 and 2 months post training | 1 month  3 months | Inoculation model Meichenbaum and Deffenbacher, 1988 | Self-report questionnaire  Archival supervisor data from police department records | Criticism self-efficacy  Perceived stress  Perceived health  Supervisor rated performance  sick days and illness duration | Significant group differences: MANOVA, F (8, 114) = 10.94, **p < 0.0001**  **Criticism self-efficacy**: F (2, 59) = 59.44, **p < 0.0001**  **Perceived stress**: F (2, 59) = 8.54, **p < 0.001**  **Perceived health**: F (2, 59) = 6.85, **p < 0.002**  **Performance evaluation**: F (2, 59) = 7.04, **p < 0.002**  No statistically significant difference in sick days or duration of illness. |
| (Gerdes et al., 2022) | USA | Randomised control trial | 92 veterans | Brief self-compassion writing task | Participants completed a writing exercise based on self-compassion elements in response to a personal negative event | Participants typed responses to guides prompts on a computer | Immediate post intervention | Not reported | Self-reported questionnaires  Respiratory sinus arrythmia (RSA)  Heart rate  Skin conductance level (SCL) | Physiological stress responses  Self-compassion  Self-criticism and affect | Significantly greater RSA during the writing task than those in the control group (F (1, 89) = 4.61, p = 0.035, η² = 0.05).  They also had significantly lower SCL reactivity compared to the control group (F (1, 89) = 4.12, p = 0.045, η² = 0.04).  No significant differences were found in heart rate.  Self-reports showed significantly greater increases in self-compassion (F (1, 89) = 12.03, p = .001, η² = .12) and lower self-criticism (F (1, 89) = 4.73, p = .032, η² = .05) in the self-compassion group. |
| (Giaume et al., 2024) | France | Nonrandomised trial | 66 Fire fighters | FIRECARE program | Based on the MBCT, supplemented by heart coherence training and positive psychology workshops. | Six weekly sessions, each lasting 2.5 hours.  Delivered by MBCT instructor with a degree in positive psychology and heart coherence training.  Remotely, in person or replaying videos. | 3 months post intervention | Mindfulness based on cognitive therapy (MBCT) | Professional quality of life scale version 5 (ProQOL-5)  Freiburg Mindfulness Inventory (FMI) | Burnout  Secondary stress  Compassion fatigue  Mindfulness | **Burnout**: 3-month follow-up, burnout scores were significantly reduced (p = 0.02)  **Secondary Stress**: Significant reduction (p = 0.003)  **Mindfulness:** Significant increase at 3-month follow-up (p = 0.05) |
| (Gon et al., 2023) | China | Randomised control trial | 54 nurses | Group training | Based on four aspects: building hope, cultivating optimism, improving self-efficacy and enhancing resilience. | Six sessions lasting 60 minutes conducted once every two weeks over a period of 3 months.  Delivered by nurse in charge. | Post intervention assessment | Psychological capital intervention model | Psychological Capital Questionnaire (PCQ-24)  Occupational benefits questionnaire  Nurse job satisfaction questionnaire (NJSQ) | Psychological capital  Occupational benefits  Job satisfaction | **Psychological Capital**: Significant improvements in the intervention group compared to the control group in hope (P = .004), optimism (P = .001  **Occupational Benefits**: Significant improvements career perception (P = .021), sense of belonging to a team (P = .040), and career benefit total score (P = .013). No significant differences in identity of relatives and friends, self-growth, or nurse-patient relationships.  **Job Satisfaction**: Significant improvements personal development (P = .001), relationships with colleagues (P = .004), the work itself (P = .003), workload (P = .036), management (P = .001), family and work balance (P = .001), and total score for job satisfaction (P = .000). No significant differences in salary and benefits. |
| (Grupe et al., 2021) | USA | Randomised controlled trial | 114 police officers | Mindfulness based training program | Mindfulness of breath and body, body scan, walking meditation, mindful movement, mindfulness of thoughts and emotions, mindful speaking and listening, eating and compassion practice. | 8 weeks training  2 hour classes and 4 hour class in week 7  Delivered by trained instructors.  Guided practices and participants encouraged to practice 6 days a week starting with 9 mins per day and increasing to 20 mins per day. | 3 months | Mindfulness based stress reduction (MBSR) | Perceived stress scale  PTSD checking or DSM-5  PROMIS anxiety  Depression  Fatigue  Sleep disturbances  Ability to participate in social roles and activities  Physical function  Pain interference  Pain intensity  Pittsburgh sleep quality inventory  Alcohol use disorders identification test  Oldbenbury burnout inventory  Health behaviours checklist  Work limitations questionnaire  Salivary cortisol  Hair cortisol  Dried blood samples for inflammation markers | Psychological distress  Mental health symptoms  Sleep quality  Cortisol awakening response  Diurnal cortisol slope  Hair cortisol concentration  Inflammatory markers | **Psychological Distress and Mental Health**: Significant reduction (p = 0.006, η² = 0.09) and at 3-month follow-up (p = 0.008, η² = 0.09). Significant improvements in PTSD symptoms (p = 0.01, d = -0.40), anxiety (p = 0.02, d = -0.34), depression (p = 0.02, d = -0.36), and fatigue (p = 0.05, d = -0.30).  **Sleep Quality**: Significant improvement in sleep quality at 3-month follow-up (p = 0.01, η² = 0.08). Significant reduction in sleep disturbances (p = 0.005, d = -0.41).  **Cortisol Awakening Response (CAR)**: Significant reduction in CAR at 3-month follow-up (p = 0.02, d = -0.33).  **Diurnal Cortisol Slope and Hair Cortisol Concentration**: No significant effects.  **Inflammatory Markers**: No significant effects on CRP, IL-6, IL-10, or TNF-α. |
| (Hsu et al., 2021) | Taiwan | Pilot study | 40 healthcare workers | Zentangle art workshop | Involves creating structured patterns to achieve a meditative state. | Workshop has five parts. Introduction to Zetangle, basic techniques, guided practice, individual creation and group sharing.  2 groups of 20, single 4 hour session. Certified Zentangle teachers delivered workshop. | Post intervention assessments | Not reported | Brief symptom rating scale (BSRS-5)  Work Stress Management effectiveness self-rating scale  General self-efficacy scale (GSES)  Workplace spirituality scale (WSS) | Psychological distress  Work related stress  Self-efficacy  Workplace spirituality | **Psychological Distress (BSRS-5)**: Significant reduction (median pre-intervention: 4.0, IQR: 2.0–6.0; median post-intervention: 4.0, IQR: 1.25–5.0; P = 0.004).  **Work-Related Stress**: Significant reduction (median pre-intervention: 38.5, IQR: 32.25–45.0; median post-intervention: 36.5, IQR: 31.0–40.0; P = 0.009).  **Self-Efficacy (GSES)**: Significant increase (median pre-intervention: 24.0, IQR: 20.25–26.0; median post-intervention: 25.0, IQR: 21.0–30.75; P = 0.010).  **Workplace Spirituality (WSS)**: Significant increase in (median pre-intervention: 97.0, IQR: 86.0–107.0; median post-intervention: 104.0, IQR: 88.0–111.75; P = 0.005). |
| (Jones et al., 2021) | United Kingdom | Nonrandomised trial | 50 police officers | Cognitive behavioural coaching (CBC) | Structured around ABCDE model of REBT  Activating Event  Belief system  Consequences  Disputation of irrational beliefs  Effective new beliefs  Aims to help participants better manage stress, improve motivation and increase satisfaction | Delivered by qualified REBC practitioner  Eight one to one sessions, each 60 minutes. | Post intervention and 6 months | Rational Emotive Behaviour Therapy (REBT) | Irrational Performance Beliefs Inventory (iPBI)  Basic Psychological Need Satisfaction in General Scale  Sport Motivation Scale (SMS)  Hair Cortisol Concentrations  Social Validation Questionnaire | Irrational beliefs  Satisfaction of basic psychological needs  Self-determined motivation  Stress | **Irrational Beliefs**: Significant reduction (Time 1: M = 92.96, SD = 9.04; Time 2: M = 79.85, SD = 10.99; Time 3: M = 72.88, SD = 16.53; p < .001).  **Basic Psychological Needs**: Significant increase (Time 1: M = 32.27, SD = 4.00; Time 3: M = 34.21, SD = 3.43; p = 0.025).  **Self-Determined Motivation**: No significant differences.  **Hair Cortisol Concentrations**: No significant differences.  **Social Validation**: Participants reported high satisfaction with the intervention (M = 6.35, SD = .10) and significant changes in their lives (M = 5.69, SD = 1.06). |
| (Jones et al., 2013) | United Kingdom | Cross sectional | 42 Armed Forced | Rest and Recuperation (R&R) | 10-14 day period of home leave during deployment. Administered as a scheduled break during deployment | Designed for rest and psychological recovery at home during deployment | Immediate post Rest and Recuperation | Not reported | GHQ-12  PLC-C  Audit | Prevenance of Common Mental Disorders (CMD)  Satisfaction with Rest and Recuperation (R&R)  Associations between Rest and Recuperation component and mental health/alcohol outcomes | No statistically significant changes in:  PTSD (PCL-C)  CMD (GHQ-12)  Alcohol use (AUDIT) |
| (Joyce et al., 2019) | Australia | Randomised control trial | 143 firefighters | Resilience@Work Mindfulness programme | Exercises included interactive exercises, audio, animations. | 6 sessions  Each session lasted 20-25 minutes  Self-paced online training programme | Baseline, post interventions (6 weeks) and 6 month follow up | Acceptance and Commitment Therapy (ACT), mindfulness-based cognitive therapy, and compassion-focused therapy. | Conor Davidson Resilience scale (CDRISC)  Brief Resilience Scale (BRS)  Freiburg Mindfulness Inventory (FMI-14)  Cognitive Fusion Questionnaire (CFQ)  Acceptance and Action Questionnaire (AAQ-II)  Self-Compassion Scale (SCS-SF)  Life Orientation Test Revised (LOT-R)  Coping Orientation to Problems Experienced (Brief-COPE)  Life Engagement Test (LET) | Resilience  Optimism  Active Coping  Emotional support  Instrumental Support  Mindfulness  Cognitive Fusion and Experiential Avoidance | **Resilience**: Significant group-by-time interaction: **P = 0.01**  At 6-month follow-up: mean increase of **1.3 points**, effect size **0.73** (95% CI: 0.38–1.06), **P = 0.002**  **BRS**: No significant difference between groups: **P = 0.09**  **Optimism** Significant improvement at 6 weeks: **P = 0.05**  **Active Coping Significant** improvement at 6 months: **P = 0.046**  **Emotional Support** Significant at 6 weeks: **P = 0.05**  **Instrumental Support** Significant at 6 weeks: **P = 0.05**  **Mindfulness** No significant change.  **Cognitive Fusion (CFQ)** and **Experiential Avoidance (AAQ-II)**: not statistically significant  Continued improvement from 6 weeks to 6 months: **P = 0.046** |
| (Judkins & Bradley, 2017) | United States | Program evaluation | 37 Armed Forces | Freedom Restoration Clinic (FRC) | Short term residential program. Included psychoeducational classes, individual therapy, military structure, physical training, sleep hygiene and nutrition | Conducted by occupational therapists with support from behavioural health professional.  Conducted on site in Afghanistan | Immediate post treatment and 30 day follow up via secure email | Not reported | Clinical records review and questionnaire | Symptom Distress  Interpersonal Relations  Social Role  Class utility  Implementation of lessons learned | **OQ-45.2 Total Score**:  Pre-treatment: Mean = 81  Post-treatment: Mean = 55  30-day follow-up: Mean = 54  Significant reduction from pre to post **p < 0.001**  No significant change from post to 30-day: **p = 0.799**  **Clinical Significance**:  73% had clinically significant symptoms at baseline (score ≥63). Reduced to 37.8% post-treatment and 35.1% at 30-day follow-up (McNemar’s test: **p < 0.001**) |
| (Khatib et al., 2022) | United States | Randomised controlled trial | 50 police departments | 3 interventions  Mindfulness based stress reduction (MBSR)  Mindfulness based resilience training (MBRT)  Mindful Performance Enhancement, awareness, and knowledge (mPEAK) | Weekly 2.5 hour sessions over 8 weeks  One 7 hour intensive session | Delivered via Zoom  Facilitated by mindfulness instructors | Pre intervention, mid intervention (4 weeks), post intervention (8 weeks) | Angry-Aggression Theory | Aggression Questionnaire (AGQ)  Perceived Stress Scale (PSS)  Beck Depression Inventory (BDI II)  Difficulties in emotion regulation scale (DERS)  State anxiety inventory (SAI)  Five facet mindfulness questionnaires (FFMQ) | Aggression  Stress  Depression  Emotion Regulation  Anxiety | Aggression – Significant reduction over time – F (2,90) = 7.88 – p < 0.001 – ηp² = 0.15 – No significant group differences  Stress – Significant reduction over time – F (2,90) = 10.01 – p < 0.001 – ηp² = 0.18  Depression – Significant reduction over time – F (2,90) = 35.00 – p < 0.001 – ηp² = 0.44  Emotion Regulation – Significant improvement – F (2,90) = 14.02 – p < 0.001 – ηp² = 0.24  Anxiety – Significant reduction – F (2,88) = 13.75 – p < 0.001 – ηp² = 0.24 |
| (Kline et al., 2024) | United States | Observational study | 282 army personnel with PTSD | Residential PTSD treatment program OASIS  Consisted of  Cognitive processing therapy (CPT)  Prolonged Exposure (PE)  Eye Movement Desensitization (EMDR)  Acupuncture  Meditation  Yoga  Nutritional support  Spiritual discussions  Physical exercise  Recreational therapy  Art music therapy  Pet assisted therapy | 10 week residential program | Delivered in person, residential setting.  Delivered by trained clinicians | Pre-treatment, weekly during treatment, post treatment assessments. | Not reported | PTSD checklist Military version (PCL-M)  Patient Health Questionnaire (PHQ-8)  Weekly self-report assessments | Residual PTSD symptoms  Residual depression symptoms  Clinically significant change defined as reduction 10 points on PCL-M | 32.6% (n = 92) experienced clinically significant PTSD symptom reduction  Depression symptom reduction:  M = −2.76, SD = 5.77  PTSD symptom reduction:  M = −18.97, SD = 8.13 |
| (Krick & Felfe, 2020) | Germany | Randomised control trial | 267 Police officers | Mindfulness and resource based worksite training | Involved mindfulness practices, body movements and cognitive education | 6 week program with weekly 2 hour sessions  Delivered by experienced trainer in classroom | Pre and post intervention over 6 weeks | Mindfulness based stress reduction principles (MBSR) | Self-reported questionnaires:  Five facet mindfulness questionnaires (FFMQ)  Health Oriented Leadership (HOL) self-care scale  Irritation scale  PNAS-X (negative affect)  Health complaints checklist  NEO-FFI and Big Five for personality traits  Custom scale for perceived norms  Physiological measures | Mindfulness  Selfcare  Psychological strain  Negative affects  Health complaints  Heart rate and variability | Mindfulness: F (1, 265) = 101.21, p < 0.001, η² = 0.28  Self-care: F (1, 265) = 228.88, p < 0.001, η² = 0.46  Psychological strain: F (1, 265) = 18.42, p < 0.001, η² = 0.07  Negative affect: F (1, 265) = 24.41, p < 0.001, η² = 0.08  Health complaints: F (1, 265) = 19.89, p < .001, η² = .07  HRV (long-term): F (1, 155) = 181.67, p < 0.001, η² = 0.55  HR (long-term): F (1, 150) = 4.13, p < 0.05, η² = 0.03  Neuroticism significantly moderated effects on mindfulness (β = 0.13, p < 0.01), self-care (β = 0.08, p < 0.05), psychological strain (β = 0.14, p < 0.05), and negative affect (β =0 .20, p < 0.001)  Openness moderated HRV (β = 0.14, p <0 .05)  Conscientiousness showed no significant moderation effects  Perceived social norm moderated mindfulness (β = 0.12, p < 0.01) and marginally moderated psychological strain (β = 0.10, p = 0.06) |
| (Krick & Felfe, 2024) | Germany | Randomised control trial | 329 Army officers | Mindfulness based intervention vs progressive muscle relaxant vs control | Involved mindfulness practices, body movements and cognitive education | 6 week program with weekly 2 hour sessions  Delivered by experienced trainer in classroom | Pre and post intervention over 6 weeks | Not reported | Self-reported questionnaires:  Five facet mindfulness questionnaires (FFMQ)  Health Oriented Leadership (HOL) self-care scale  Irritation scale  PNAS-X (negative affect)  Health complaints checklist  NEO-FFI and Big Five for personality traits  Custom scale for perceived norms  Physiological measures:  Heart rate  Heart rate variability | Mindfulness  Self-care  Positive affect  Psychological strain  Health complaints  Heart rate and variability | Mindfulness group showed significantly greater improvements than Progressive muscle relaxant and control in:  Mindfulness: F (2, 209) = 28.68, p < 0.001, η² = 0.15  Positive affect: F (2, 209) = 24.94, p < 0.001, η² = 0.13  Self-care: F (2, 209) = 20.24, p < .001, η² = 0.11  Health complaints: F (2, 209) = 27.61, p < .001, η² = .14  Both MBI and PMR reduced psychological strain: F (2, 209) = 15.42, p < .001, η² = .09  Heart rate variability increased significantly in MBI group:  Pre–post intervention: F (2, 209) = 37.88, p < .001, η² = .27  Session 6: F (2, 209) = 20.53, p < .001, η² = .16  HR decreased significantly in MBI group:  Pre–post intervention: F (2, 209) = 8.77, p < .001, η² = .08  Session 6: F (2, 209) = 19.23, p < .001, η² = .16  PMR showed no significant changes in HRV or HR |
| (Leggett et al., 2013) | United States | Pilot study | 13 nurses | Educational intervention on moral distress | 4 weekly sessions lasting 60 minutes.  Definitions and concepts of moral distress  Signs and symptoms  Case discussions  Ethical issues e.g. end of life care  Strategies for coping with moral distress | Conducted in person at place of work | Post intervention and 6 weeks after intervention | Qualitative interviews from nurses | Moral distress scale (MDS-R)  Self-efficacy scale (SE) | Moral distress | At 6-week follow-up:  MDS-R scores: Group B median = 69.0, Group A median = 60.5  U = 23, z = 0.268, P = 0.775 (not significant)  SE scores: Group B median = 33.0, Group A median = 36.5  U = 32, z = 1.58, P = 0.114 (not significant) |
| (Leonard & Alison, 1999) | Australia | Nonrandomised control trial | 60 police officers | Critical Incident stress debriefing (CISD) | Phases include introduction, facts, thoughts, emotions, assessment, education, and re entry | Conducted within 72 hours post shooting incident  One time group session facilitated by trained professionals | Post incident | 7 phase model by Mitchell and Bray (1990) | Questionnaire  Coping Scale  State trait anger expression inventory (STAXI) | Coping strategies  Anger levels | Coping:  CISD group scored higher on:  Active coping: F = 4.50, p < 0.05  Positive reinterpretation and growth: F = 7.26, p < 0.01  No significant difference in total adaptive or maladaptive coping scores  Anger:  CISD group had significantly lower scores on:  State anger: F = 5.34, p < 0.05  Trait anger: F = 10.64, p < 0.01  Angry temperament: F = 6.68, p < 0.05 |
| (Mackintosh et al., 2017) | United States | Randomised control trial | 58 military veterans | RELAX mobile app and Anger Management (AMT) group sessions | Mobile app with wearable heart rate monitor, remote server and therapist web interface | AMT sessions twice weekly for 6 weeks  Relax app used in-between  Delivered by doctoral and master level therapists | Baseline, post treatment, 3 months, 6 months | Anger management treatment (AMT) | STAXI-2  Dimensions of Anger (DAR-5)  PTSD checklist for DSM-5  Patient health questionnaire  Inventory of psychosocial functioning  Technology feedback questionnaire | Anger  PTSD symptoms  Depression  Interpersonal functioning  Engagement | No significant differences between AMT and AMT + RELAX on primary or secondary outcomes  Both groups showed significant reductions in anger:  STAXI-Trait: d = 1.00 (mean across time points)  STAXI-AEI: d = 1.07  DAR-5: d = 0.72  PTSD symptoms (PCL-5): significant reduction (t = −2.51, p = 0.01, d = 0.33)  Depression (PHQ-9): marginal reduction (t = −1.76, p = 0.08, d = 0.35)  No significant change in interpersonal functioning (B-IPF) |
| (Maguire et al., 2024) | United States | Pilot Study | 128 police officers | 22ZERO Trauma Resilience | Non trauma based, non-exposure therapy  Focuses on emotional regulation through guided imagery and parasympathetic activation | Conducted online via telehealth  Delivered by trained providers | Before and Immediate post treatment | no trauma-based PTSD treatment | Questionnaires  PTSD checklist (PCL-5)  Generalised anxiety disorder 7 item scale (GAD-7)  Patient Health Questionnaire (PHQ-9) | PTSD symptoms  Anxiety  Depression | Anxiety (GAD-7, n = 118):  t (117) = 20.3, p < 0.001  Cohen’s d = 1.87 (Hedges’ g = 1.86)  Depression (PHQ-9, n = 82):  t (81) = 13.8, p < 0.001  Cohen’s d = 1.52 (Hedges’ g = 1.52)  PTSD  Pretest mean = 43.4, SD = 16.2  Post-test mean = 7.3, SD = 11.2  t (127) = 23.69, p < 0.001  Cohen’s d = 2.09 (Hedges’ g = 2.09) |
| (Mainsbridge et al., 2020) | Australia | Pilot study | 43 desk based employee from police and emergency management department | Movement Microbreaks via computer software | Software installed on work on computers prompted hours microbreaks  Engage in self-selected low intensity, short duration non exercises physical activity | 13 weeks | Post 13 weeks  Washout assessment 26 weeks | National guidelines for office employees | Police stress questionnaire (PSQ-Org)  Profile of Mood States (POMS)  Online questionnaire | Perceived organisational stress  Mood dates | Significant group × time interaction: F (2,78) = 4.21, p = 0.02, η² = 0.10  No significant group × time interactions for POMS-Fatigue: F (2,78) = 1.39, p = 0.25, η² = 0.04 |
| (Maloney et al., 2024) | United States | Observational study | 43 emergency doctors  23 paramedics  17 nurses | EMS Code Lavendar Programme | Peer support initiative | Delivered by supervisors, educators, medical directors | 20 months  pre and post programme | Not reported | Professional quality of life scale (ProQOL-5)  Abbreviated Maslach Burnout Inventory (aMBI)  Surveys | Changes in quality of life  Changes in burnout | No significant changes in ProQOL-5 or aMBI subscale scores from pre- to intraprogram:  Compassion satisfaction: 36.7 (p = 0.62)  Secondary traumatic stress: 21.6 (p = 0.22)  Burnout: 26.0 (p = 0.98)  Emotional exhaustion: 8.1 (p = 0.56) |
| (Markwell et al., 2016) | United states | Cross sectional | 210 nurses | Snack and relax program (S&R) | Monthly sessions offering  Healthy snacks  Holistic relaxation modalities (Reiki, healing touch, massage therapy, Jin Shin Jyutsu) | Delivered by certified volunteer practitioner  Held in the hospital, sessions lasted 10-15 minutes | Pre/post evaluation  Survey after 5 sessions | Not reported | Self-reported stress  Blood pressure  Heart rate  Respiratory rate  ProQOL | Physiological and psychological effects of snack and relax  Quality of life | Self-reported stress: 3.0 (p < .001)  Respiratory rate: 16.8 (p = .001)  Heart rate: 72.3 bpm (p < .001)  Blood pressure: No significant change  **ProQOL Survey**  Low Compassion Satisfaction (CS): 28.5%  High Burnout: 25.3%  High Secondary Traumatic Stress (STS): 23.4%  No significant differences in CS, Burnout, or STS between S&R participants and nonparticipants  Significant findings:  Non-Caucasian nurses had higher CS than Caucasians (p = 0.008)  Nurses in surgical services had lower burnout than those in intensive/telemetry care (p = 0.015) |
| (McCall, 2023) | United states | Observational | 13 air medical crew members | Peer support program | Outreach after emotionally challenging transport via text or phone call  Referral initiated by peer or via electronic desktop link | Training provided by clinical social worker | 16 months | Not reported | Professional quality of life scale (ProQOL) | Quality of life | No significant differences between those contacted vs. not contacted by peer support  **Longitudinal Comparison**  No statistically significant changes:  Compassion Satisfaction: 37.08, p = .294  Secondary Trauma Stress: 22.69, p = .376  Burnout: 22.38, p = .602 |
| (McKeon et al., 2023) | Australia | Stepped wedge cluster trial | 90 participants including paramedics, fire fighters, police officers, | Physical activity and diet program | Two weekly group video calls via zoom  Fitbit devices | Delivered via Facebook groups  Facilitated by exercise physiologists, dietician, peer facilitator | 1 month post intervention | behaviour change techniques including fostering social support, self- monitoring and shaping knowledge | Kessler-6 (K6)  Depression, Anxiety and stress scale (DASS-21)  PTSD checklist (PCL-5)  Assessment of quality of life (AQoL-6D)  Social support and exercise  Suicidal ideation attributes scale (SIDAS)  Physical activity vital signs (PAVS) | Psychological distress  Depression  Anxiety  PTSD  Physical activity  Sedentary time  Sleep quality  Quality of life  Social support  Suicidal ideation | Psychological distress significant reduction during first 6 weeks of intervention (b = −0.441, p < 0.001). Plateaued thereafter (b = −0.009, p = 0.883)  Secondary outcomes (pre–post):  Anxiety: p = 0.02, Cohen’s d = 0.30  Stress: p = 0.003, d = 0.22  Quality of life: p = 0.001, d = 0.26  MVPA: p = 0.03, d = 0.29  Walking time: p = 0.002, d = 0.43  Sedentary time: p = 0.045, d = 0.25  Family support to exercise: p = 0.004, d = 0.45  PTSD symptoms (PCL-5): p = 0.001, d = 0.23  No significant changes in depression (p = 0.15), sleep quality (p = 0.07), suicidal ideation (p = 0.45), or friend support to exercise (p = 0.19) |
| (McLean, Cook, et al., 2024) | United States | Mixed methods | 29 military personal | Web Based Prolonged Exposure Therapy (web-PE) | Comparing Web-PE to in-person present-centred therapy (PCT) | 10 sessions, self-guided, therapist facilitated online program  Included breathing retraining, psychoeducation, in vivo and imaginal exposure and homework review | Post treatment | Prolonged exposure therapy | Perceptions of Web-PE Questionnaire (PWPQ)  Telephone interview  PTSD checklist (PCL-5) | PTSD symptom change | **64%** experienced a **≥10-point reduction** in PCL-5 scores. |
| (Meland et al., 2015) | Norway | Nonrandomised trial | 40 army personnel | Mindfulness Training (MT) | Tailored to fit a high performance military environment | Delivered in person over 4 month period  Delivered by instructors with 10 years or more of meditative practice and formal accreditation | Post test evaluation | Mindfulness stress reduction (MBSR) program | Salivary cortisol  Sustained Attention to Respond task (SART)  Attentional Capture Task (ACT)  Five Facet Mindfulness Questionnaire  Norwegian Sports Anxiety Scale (SAS-n)  Hopkins Symptom Checklist  NASA-TLX  Sleep logs | Cortisol slopes  Attention control  Self-perceived mindfulness  Anxiety  Depression  Sleep quality and duration  Positive response outcome expectancy | Cortisol increased to 8.3 nmol/l; Control group decreased from to 9.8 F (1,38) = 5.537, p = .024, η² = 0.127  Cortisol Awakening Response: No significant change  Reaction Time: MT group increased to 322 ms; Control group decreased from 326 to 303 F (1,38) = 8.202, p = 0.007, η² = 0.174  Mental Demand: MT group decreased to 11.25; Control group remained nearly unchanged F (1,38) = 10.805, p = 0.002, η² = .231  ACT: MT group increased from to 1.40 F (1,38) = 4.878, p = .033,  (FFMQ): MT group increased to 3.21 p < .05  Depression: Both groups decreased F (1,38) = 5.837, p = 0.022, η² = 0.154  Worry: Both groups decreased F (1,38) = 5.984, p = .020, η² = .158  Sleep Length: Both groups increased F (1,38) = 5.971, p = .020, η² = .162  Sleep Quality: Both groups increased F (1,38) = 4.910, p = .034, η² = .002 |
| (Millegan et al., 2021) | United States | Cohort study | 239 Navy and Marine Corps | Mind-Body Medicine (MBM) program | Include mindfulness, meditation, yoga, cognitive restructuring, sleep hygiene and social support | 7 week program  Delivered in classroom setting by healthcare staff including psychologists, social workers, nurse practitioners | Pre and post intervention | Adapted from the Benson Henrys Institutes Stress Management and Resiliency Training Program | Perceived stress scale (PSS)  Brief Pain Inventory (BPI)  Response to stressful experience scale (RSES)  Patient Health Questionnaire (PHQ-8)  Generalised Anxiety Disorder (GAD-7)  PTSD Checklist Specific (PCL-S)  Pittsburgh Sleep Quality Index (PSQI)  Sheehan Disability Scale (SDS)  WHOQOL BREF | Stress  Pain  PTSD symptoms  Sleep quality  Functional impairment  Anxiety  Depression  Quality of life  Knowledge and practice of mind body techniques | Perceived Stress Scale: t (132) = 7.69, p < .001, d = 0.667  PTSD Checklist: t (225) = 3.30, p < .001, d = 0.220  Sheehan Disability Scale: t (231) = 6.51, p < .001, d = 0.428  Pittsburgh Sleep Quality Index: t (235) = 5.57, p < .001, d = 0.363  Generalised Anxiety Disorder-7: t (237) = 5.03, p < .001, d = 0.326  PHQ-8 (Depression): t (238) = 6.92, p < .001, d = 0.448  WHOQOL-BREF Physical Health: t (236) = 5.94, p < .001, d = 0.386  WHOQOL-BREF Psychological Health: t (238) = 6.94, p < .001, d = 0.449  Response to Stressful Experiences Scale: t (85) = 6.35, p < .0017  Brief Pain Inventory: t (108) = 4.41, p < .0017  Perceived Stress Scale: t (46) = 3.09, p = .003 (not significant after Bonferroni correction)  Mind-body practice and knowledge  Comfort with meditation: χ² (2, N = 144) = 36.63, p < .001  Understanding of meditation techniques: χ² (2, N = 146) = 76.41, p < .001  Weekly practice increased from M = 1.12 to M = 3.61 days/week, t (226) = 15.47, p < .001 |
| (Mohr et al., 2024) | United states | Randomised control trial | US Army  Leaders n=99  Service members n=276 | Supportive leadership program | Focused on proactive and responsive supportive behaviours, including emotional support and destigmatising mental health | 90 minute hybrid training (30 minutes in person, 30 minutes computer based, 30 minutes in person)  Led by trained facilitators following a standardised script | Baseline and 4 months post baseline | previous supportive-supervisor training interventions (e.g., Hammer et al., 2011; 2021) and mental health awareness training for managers (e.g., Dimoff et al., 2016; Dimoff & Kelloway, 2019) and the empirically and theoretically informed framework introduced for Mental Health Supportive Supervisor Behaviours (Hammer et al., 2024). | Online surveys  Brief loneliness scale  Team cohesion scale  Emotional support subscale  Supervisor destigmatising behaviour scale | Loneliness  Unit belonging  Perceived supervisor emotional support  Perceived supervisor destigmatising behaviour | Loneliness: Significantly lower loneliness at 4 months b = −0.46, SE = 0.23, p < .05, β = −0.31, d = −0.21  Unit belonging: No significant main effect b = −0.01, SE = 0.18, p > .05  Supervisor emotional support: No significant main effect b = −0.05, SE = 0.08, p > .05  Supervisor destigmatising behaviour: No significant main effect b = −0.06, SE = 0.10, p > .05  Significant interaction effects for:  Supervisor emotional support: b = 0.07, SE = 0.03, p < .05, β = 0.10  Supervisor destigmatising behaviour: b = 0.09, SE = 0.03, p < .01, β = 0.13 |
| (Morland et al., 2016) | United States | Pilot study | 5 military personnel | Remote Exercises for Learning Anger and Excitation Management (RELAX) mobile app | The app features anger monitoring, biofeedback and exercises such as time out plans, progressive muscle relaxation, diaphragmic breathing and cognitive restructuring | Used alongside a cognitive anger management protocol  Veterans completed assignments using the app | 3 months post treatment | Anger management therapy | State Trait Anger Expression Inventory (STAXI-2)  Dimensions of Anger Reactions (DAR-5)  Inventory of Psychosocial functioning brief (B-IPF)  Patient Health Questionnaire (PHQ-9)  PTSD checklist for DSM (PCL-5) | Anger  PTSD symptoms  Depression symptoms  Psychosocial functioning | STAXI-Trait Baseline: M = 26.0 (SD = 6.7), Median = 23.0 Post-treatment: M = 15.3 (SD = 3.8), Median = 15.0, p < 0.05 3-month follow-up: M = 19.5 (SD = 11.2), Median = 16.5  STAXI-Anger Expression Index Baseline: M = 54.0 (SD = 18.2), Median = 55.5 Post-treatment: M = 20.0 (SD = 15.1), Median = 20.5, p < 0.10 3-month follow-up: M = 31.5 (SD = 25.0), Median = 25.0  PCL-5 (PTSD symptoms) Baseline: M = 66.3 (SD = 7.7), Median = 64.5 Post-treatment: M = 35.8 (SD = 30.9), Median = 35.5, p < 0.10 3-month follow-up: M = 35.3 (SD = 32.4), Median = 31.5, p < 0.10  PHQ-9 (Depression) Baseline: M = 20.3 (SD = 10.0), Median = 16.0 Post-treatment: M = 15.3 (SD = 13.6), Median = 13.0 3-month follow-up: M = 7.3 (SD = 10.0), Median = 3.5, p < 0.05  B-IPF (Psychosocial Functioning) Baseline: M = 2.9 (SD = 1.4), Median = 3.4 Post-treatment: M = 2.2 (SD = 1.9), Median = 2.9 3-month follow-up: M = 1.6 (SD = 1.6), Median = 1.3, p < 0.05  DAR-5 (Anger Reactions) Baseline: M = 18.3 (SD = 4.2), Median = 20.0 Post-treatment: M = 10.0 (SD = 3.9), Median = 10.5, p < 0.01 3-month follow-up: M = 14.0 (SD = 6.8), Median = 12.5 |
| (Márquez et al., 2021) | Spain | Pilot study | 20 police officers | Mindfulness Based Intervention (MBI) | Included individual and group practices. Conscious movement, breathing meditation, observation of bodily sensations, emotions and thoughts | 7 week program (six 2 hour sessions and one 4 hour session)  Delivered in person | Post intervention | Mindfulness based stress reduction (MBSR) | Five Facets Mindfulness Questionnaire (FFMQ)  Self-Compassion Scale (SCS)  Professional Quality of Life Scale (ProQOL)  Perceived Stress Scale (PSS)  Qualitative feedback questionnaire | Mindfulness  Self-compassion  Compassion satisfaction  Compassion fatigue  Burnout  Perceived stress | **Mindfulness** (MANOVA: F (5,14) = 7.441, p = 0.001, η² = 0.727)  **Self-Compassion** No significant overall change (MANOVA: F (6,13) = 1.001, p = 0.465, η² = 0.316)  **Compassion Satisfaction** F (1,19) = 5.929, p = 0.025, η² = 0.238  **Compassion Fatigue** No significant change F (1,19) = 0.044, p = 0.836, η² = 0.002  **Burnout** No significant change F (1,19) = 0.040, p = 0.843, η² = 0.002  **Perceived Stress** Significant reduction t (18) = 2.863, p = 0.010, Cohen’s d = 0.611 (medium effect size) |
| (Narayanan et al., 2024) | New Zealand | Pilot Study | 5 surgeons | Surgeon selected background music played during surgery | Music was played through a speaker at a low medium volume during surgery. | Genre and playlist were chosen by the primary operator or delegated to a team member.  Music could be adjusted or turned off upon request. | Immediate post-surgery | Not reported | Heart rate variability (HRV)  Six item state trait anxiety inventory (STAI-6)  Surgical task load index (SURG-TLX) | Physiological stress  Psychological stress and workload | Significant intraoperative changes from baseline across all cases:  Mean heart rate increased at midpoint estimated marginal mean difference (EMMD) +4.4 bpm, P < 0.001) and endpoint (EMMD +3.0 bpm, P < 0.01)  Root mean square of successive differences (RMSSD) decreased at start (EMMD −2.09, P < 0.001) and end (EMMD −1.40, P < 0.001)  PNS index decreased (EMMD −0.12, P < 0.001)  Baevsky’s stress index increased (EMMD +0.95, P = 0.031) No significant differences between music and control arms in most HRV parameters, except:  Low frequency (LF) /High Frequency (HF) ratio was slightly lower in the music group at the end of operation (13.2 vs. 11.6, P = 0.03)  Psychological Outcomes  STAI-6 scores: No significant difference between music (mean 8.6) and control (mean 8.9), P = 0.39  SURG-TLX total score: No significant difference between music and control (mean 48 ± 22), P = 0.59  No significant differences in SURG-TLX subscales (e.g., distractedness, mental workload) |
| (Nassif et al., 2023) | United states | Randomised control trial | 1584 army personnel | Combined mindfulness and yoga program | Mindfulness based attention training (MBAT) 15 minute group mindfulness practices and embedded mindfulness exercises  Yoga consisted of 30 minutes of hatha yoga | 4 week program.  Mindfulness training delivered by trained performance experts.  Yoga led by certified instructors. | Four time points baseline (T1), week 4 (T2), week 6 (T3) and week 9 (T4) | Not reported | Patient health questionnaire (PHQ-2)  Generalised anxiety disorder (GAD2)  Insomnia severity index | Depression  Anxiety  Sleep problems | Depression Positive depression decrease (−12.6%)  b = −0.18, SE = 0.07, p = 0.028, OR = 0.83 [95% CI: 0.71, 0.98]  Anxiety Positive screens decreased over time across all groups, but no significant time-by-condition interaction b = 0.09, SE = 0.09, p = 0.273  Sleep Problems decreased in the intervention group (−1.4%)  b = −0.68, SE = 0.16, p = 0.027, OR = 0.51 [95% CI: 0.28, 0.93] |
| (Navarrete et al., 2022) | Spain | Pilot study | 38 police officers | Mindfulness Aplicado al Bienestar Policial  (Mindfulness to promote police wellbeing) | Co designed by psychologist and police officer  Based on mindfulness practices targeting attention regulation, body awareness, emotion regulation and self-perspective.  Participants also completed daily home practice and weekly emotion assessments | 8 week program  Delivered by psychologist and police officer in person sessions | Pre and post intervention  Weekly emotion tracking during the 8 week sessions | Not reported | Five Facets of Mindfulness Questionnaire (FFMQ-SF)  Self-compassion scale (SCS-SF)  Depression, Anxiety and Stress Scale (DASS-21)  Frequency of suicidal ideation inventory (FSII)  PROMIS Sleep Disturbance (PROMIS-SD)  Copenhagen Burnout Inventory (CBI)  Difficulties in emotion Regulation Scale (DERS-SF)  Discrete Emotions Questionnaire (DEQ) | Mindfulness  Self-compassion  Depression  Anxiety  Stress  Suicidal ideation  Sleep quality  Burnout  Emotion regulation  Weekly emotional stress | Mindfulness (FFMQ-SF) significant increase, t (19) = -3.81, p = .001, η² = 0.43. Between-group ANCOVA: F (1,33) = 5.90, p = .021, ηp² = 0.15  Self-Compassion (SCS-SF) significant increase, t (19) = -3.77, p = .001, η² = 0.43. Between-group ANCOVA: F (1,33) = 5.77, p = .022, ηp² = 0.15  Depression (DASS-21). significant decrease, t (19) = 4.74, p < .001, η² = 0.54. Between-group ANCOVA: F (1,33) = 9.70, p = .004, ηp² = 0.23  Anxiety (DASS-21). significant decrease, t (19) = 4.05, p = .001, η² = 0.46. Between-group ANCOVA: F (1,33) = 7.31, p = .011, ηp² = 0.18  Stress (DASS-21) significant decrease, t (19) = 4.47, p < .001, η² = 0.51. Between-group ANCOVA: F (1,33) = 4.51, p = .041, ηp² = 0.12  General Distress (DASS-21 total) significant decrease, t (19) = 5.65, p < .001, η² = 0.63. Between-group ANCOVA: F (1,33) = 10.54, p = .003, ηp² = 0.24  Suicidal Ideation (FSII) decrease, t (19) = 2.33, p = .031, η² = 0.22. Between-group ANCOVA: not significant, p = .455, ηp² = 0.02  Sleep Quality (PROMIS-SD). significant improvement, t (19) = -5.04, p < .001, η² = 0.57. Between-group ANCOVA: F (1,33) = 6.99, p = .012, ηp² = 0.18  Burnout (CBI) Personal burnout: t (19) = 4.13, p = .001, η² = 0.47; ANCOVA: F (1,33) = 7.99, p = .008, ηp² = 0.20  Work-related burnout: t (19) = 2.90, p = .009, η² = 0.31; ANCOVA: F (1,33) = 7.23, p = .011, ηp² = 0.18  Emotion Regulation (DERS-SF) Total score: t (19) = 3.25, p = .004, η² = 0.36  Weekly Emotional States (MBI group only) Significant decreases in anger (p = .005), disgust (p = .009), anxiety (p < .001), sadness (p < .001), and desire (p < .001) over 8 weeks  No significant changes in fear, relaxation, or happiness |
| (Niemeyer et al., 2020) | Germany | Randomised control trial | 37 Army personnel | Therapist guided internet based cognitive behavioural therapy (iCBT) | 3 phases  Biographical reconstruction  Exposure to traumatic event  Cognitive restructuring  Written communication with licensed therapists. Optional phone support for technical issues. | 5 week course, 10 structured writing assignments.  Online based platform.  Therapist provided feedback within one working day on written assignments. | 3 months post treatment | Prolonged exposure and cognitive restructuring | Clinician administered PTSD scale (CAPS-5)  Generalised Anxiety Disorder Scale (GAD-7) | PTSD severity  Anxiety symptoms | CAPS-5 (PTSD symptoms) −5.42, p = .037, d = −0.42  GAD-7 (Anxiety symptoms)  Pre-to-follow-up predictive mean mapping (MI-PMM) = −3.04, p = .020, d = −0.58  Pre-to-follow-up= −2.20, p = .005, d = −0.51 |
| (Nimenko & Simpson, 2014) | United Kingdom | Pilot study | 24 army personnel | Group activity psychological decompression (GAPD) | Archaeological digs on Salisbury Plain  Activities included excavation, artifact sorting and informal social interaction | 5 consecutive days  Supervised by professional archaeologists and military medical staff | Immediate post intervention | Not reported | PHQ-9  GAD-7  Work and Social Adjustment Scale (WSAS)  AUDIT  Impact of events scale revised (IES-R) | Depression  Anxiety  Social functioning  Alcohol use  PTSD symptoms | WSAS mean change -2.6  PHQ-9 mean change -3.4  GAD-7 mean change -3.3  AUDIT mean change -1.1  IES-R mean change -1.1 |
| (Nwokeoma et al., 2019) | Nigeria | Randomised control trial | 63 police officers | Rational Emotive Occupational Health Coaching (REOHC) | Coaching program  Topics included  Understanding stress  Identifying stressors  Cognitive restructuring  Behavioural skills  Stress management techniques | 12 weeks,  60 minute weekly sessions  Help in person  Delivered by trained facilitator | 3 months post intervention | Rational Emotive Behaviour Therapy (REBT) Principles | Organisational Police Stress Questionnaire (PSQ-Org)  Operational Police Stress Questionnaire (PSQ-Op) | Work related stress management | reduction in organizational stress (F (1,60) = 550.791, p < .001, η² = .902)  reduction in operational stress (F (1,60) = 37.232, p < .001, η² = .383) |
| (Onyishi et al., 2021) | Nigeria | Randomised control trial | 153 police officers | Rational Emotive Occupational Health Coaching (REOHC) | Structured coaching programme  Included  Cognitive, behavioural and emotional techniques  Relaxation training  Self-coaching strategies | Weekly 2 hour sessions | 3 months post test | Rational Emotive Behaviour Therapy (REBT) | Life satisfaction scale (LS)  Positive/negative affect scale (SPANE-B)  Flourishing Scale (FS)  Perceived Work Ability Index (PWAI) | Subjective wellbeing  Perceived worked ability | At 3-month follow-up:  Life satisfaction: F (1,148) = 25.78, p < .001, η² = .576  Affect balance: F (1,148) = 41.85, p < .001, η² = .934  Flourishing: F (1,148) = 13.07, p < .001, η² = .537  Perceived work ability: F (1,148) = 18.45, p < .001, η² = .752 |
| (Otis et al., 2024) | United States | Randomised control trial | 95 army personnel | Surf therapy and hike therapy as adjunctive treatments to standard care for major depressive disorder (MDD) | Surf therapy conducted at a public beach  Hike therapy at various San Diego County locations | In person group session 6 week programs with weekly 3-4 hour sessions  Led by master’s level recreation therapists and exercise physiologists | 3 month post intervention | Not reported | Mini international neuropsychiatric interview MINI-7  Montgomery-Asberg Depression Rating scale MADRS  Patient health questionnaire PHQ-9  Patient health questionnaire PHQ-4  PTSD checklist PCL-5  International physical activity questionnaire IPAQ-SF | Depression symptom severity  PTSD symptom severity | MADRS (MD = −6.49, p < .001)  PHQ-9 (MD = −4.50, p < .001)  No significant difference in depression improvement between MDD-only and MDD-PTSD groups (p = .247–.349)  No significant change from post program to 3-month follow-up (MADRS: p = .223; PHQ-9: p = .105) |
| (Pallavicini et al., 2022) | Italy | Observational | 20 healthcare professionals  11 doctors, 9 nurses | MIND VR | Virtual reality based psychoeducational experience  Immersive experience designed to educate users about stress and anxiety | 15 minutes to complete each path, definitions, causes/symptoms and treatments | Immediately after intervention | Emotional design  User centre design | System usability scale (SUS)  Net promoter score (NPS)  Slater usoh steed presence questionnaire (SUS-II)  Igroup presence questionnaire (IPQ)  Ad hoc learning questionnaire  Visual Analogue Scale for Emotions (VAS-E)  State trait anxiety inventory (STAI-Y1)  Semi structured interviews | Usability  Emotional response  Anxiety  Presence  Learning effectiveness | Happiness (VAS-HP): 68.5 (t (19) = 2.59, p = 0.018)  Fear (VAS-FE): 9.7 (t (19) = 2.57, p = 0.019)  Sadness (VAS-SD): 10.7 (t (19) = 2.33, p = 0.031)  (STAI-Y1): 32.5 (t (19) = 2.65, p = 0.016)  SUS-II: M = 4.1, SD = 1.1  IPQ-G (general presence): M = 5.1, SD = 1.7  PQ-SP (spatial presence): M = 4.2, SD = 1.2  IPQ-INV (involvement): M = 3.3, SD = 1.1  IPQ-REAL (realism): M = 3.3, SD = 1.1 |
| (Peng et al., 2024) | China | Randomised control trial | 1,399 emergency medical first responders (doctors and nurses) | Psychological First Aid (PFA) training programme | Included culturally relevant examples, case studies and role play.  Six modules covering core concepts, knowledge and practice | Delivered using a training of trainers manual and johns Hopkins university PFA guidelines | 1 day, 1 month and 2 months post intervention | WHO guidelines for Psychological first aid | PFA skills, knowledge and attitude (PFA SKA)  Attitude questionnaire  Post traumatic growth index (PTGI)  General self-efficacy scale (GSE)  Professional quality of life scale (ProQOL-5) | PFA SKA score  Post traumatic growth  Self-efficacy  Professional quality of life | **General Self-Efficacy (GSE):** aMD = 0.845 95% CI: 0.0716 to 1.618 p = 0.032 Cohen’s d = 0.20 (small effect)  **Post-Traumatic Growth Index (PTGI):** aMD = 2.114 95% CI: −0.500 to 4.728 p = 0.113 Cohen’s d = 0.24 (not statistically significant)  **Professional Quality of Life (ProQOL):** aMD = 1.509 95% CI: −1.233 to 4.252 p = 0.281 Cohen’s d = 0.04 (not statistically significant) |
| (Peterson et al., 2023) | United States | Randomised control trial | 234 military personnel | Comparison of two formats of Prolonged Exposure (PE) therapy  Massed PE  Intensive outpatient program PE (IOP-PE) | Include  Psychoeducation  Diaphragmatic breathing  In vivo and imaginal exposure  Trauma processing  IOP-PE had additional components tailored to PTSD | Delivered by trained therapists  Massed PE 15 daily 90 minute sessions over 3 weeks  IOP-PE 15 full day sessions over 3 weeks with 8 treatments | Baseline, during treatment, 1,3,6 month follow up | Prolong Exposure Therapy | PTSD scale (CAPS-5)  PTSD checklist (PCL-5)  Sheehan disability scale (SDS)  Brief inventory of psychological functioning (B-IPF)  Adverse even monitoring | PTSD severity  PTSD remission  Reliable change index  Functional impairment | Symptom Maintenance (1- to 6-month follow-up):  CAPS-5:  IOP-PE intervention: −1.23 (95% CI: −3.72 to 1.27), P = .33  Massed-PE intervention: +3.21 (95% CI: 0.65 to 5.77), P = .01  PCL-5:  IOP-PE intervention: −0.21 (95% CI: −3.47 to 3.06), P = .90  Massed-PE intervention: +3.02 (95% CI: −0.36 to 6.40), P = .08 |
| (Price et al., 2022) | Canada | Experimental | 215 firefighters and paramedics | Critical incident stress management (CISM) peer support | Includes  Assessment and triage  Strategic planning  Individual crisis intervention  Informational group crisis information  Interactive group crisis intervention  Resiliency training | Delivered by trained peer supporters and overseen by registered mental health provider | After intervention | Not reported | Peer support survey  Alcohol use disorders and identification test (AUDIT)  Generalised anxiety disorder (GAD-7)  Panic disorder severity scale (PDSS-SR)  Patient health questionnaire (PHQ-9)  PTSD checklist (PCL-5)  Social interaction phobia scale (SIPS) | Prevenance of mental disorders  Perception of CISM  Association between CISM fidelity and mental health outcomes | Perceptions of CISM (Experimental Group Only): No significant differences in perceived skills, use, or value of CISM by gender, age, or years of service  PTSD significantly associated with lower perceived skills from CISM (F (6, 57) = 3.33, p = 0.007, R² = 0.259; PTSD: t = 2.14, p = 0.037) |
| (Rajeswari et al., 2020) | India | Experimental | 120 nurses | Accelerated Recovery Program (ARP) | Guided imagery  Neurolinguistic programming (NLP)  Thought Field Therapy (TFT)  Self-management planning  Self-analysis | Once a week session for 5 week s  Each session lasted 90-120 minutes | Post intervention 5^th^ week, 3^rd^, 6^th^, 9^th^ and 12 months | Not reported | Professional quality of life scale (ProQOL)  Included compassion satisfaction (CS)  Burnout (BO)  Secondary traumatic stress (STS) | Changes in  Compassion Satisfaction  Burn out  Secondary traumatic stress | Compassion Satisfaction (CS) Repeated Measures ANOVA: F (1,118) = 120.10, p < 0.001Burnout (BO) Repeated Measures ANOVA: F (1,118) = 123.11, p < 0.001Secondary Traumatic Stress Repeated Measures ANOVA: F (1,118) = 205.18, p < 0.001 |
| (Ramey et al., 2016) | United states | Pilot study | 38 police officers | Resilience training program | Based on self-regulation of emotional and physiological responses to stress  Topics included physiology of stress  Stress triggers  Breathing and heart rate regulation  Positive emotion focus to improve decision making | One 2 hour class and one telementor session  Delivered by a research team member and psychologist | Data collected at baseline, 3 months and 6 months | Based on interventions previously successful in the military | Heart rate variability (HRV)  Blood pressure  BMI  Blood tests hbA1c, LDL, cholesterol, Triglyceride, CRPS  Perceived stress scale (PSS)  Vital exhaustion  Impact of events scale  Response to stressful experience scale  Personal and organisational quality assessment | Psychological stress  HRV  Cardiovascular risk factors  Coherence | HbA1c: decreased by 0.1 (SD = 0.3), P = 0.02, Cohen’s d = 0.42  Impact of Events Scale (Avoidance): decreased by 2.4 (SD = 8.0), P = 0.09, d = 0.29  Anger and Resentment: decreased by 0.2 (SD = 0.7), P = 0.08, d = 0.30  Emotional Vitality: decreased by 0.1 (SD = 0.8), P = 0.57, d = 0.09  HRV (High Frequency - parasympathetic):  Workday: increased by 2.6 (SD = 5.9), P = 0.03, d = 0.43  Off-day: increased by 2.5 (SD = 5.5), P = 0.02, d = 0.46  HRV (Low Frequency - sympathetic): decreased accordingly  RMSSD (off-day): increased by 5.6 (SD = 14.4), P = 0.04, d = 0.39  Coherence: increased by 38.5% (SD = 21.9), P < 0.001  Correlated with reduced HbA1c (r = –0.66, P < 0.001)  Correlated with reduced organizational stress (r = –0.44, P = 0.03) |
| (Ranta, 2012) | India | Experimental | 80 police officers | Multidimensional psychological intervention | Stress management and relaxation training  Self-management and mood management techniques  Rehearsal of all phases and application in imaginary situations during mediation | Small groups through workshops | Immediate post intervention | Indian psychological technique | Police stress questionnaire created by author  Hindi version of anger expression scale (AX/EX) | Job stress  Anger in  Anger out  Anger control | Job Stress: F (1,72) = 8.046, p < 0.001 Pre-treatment means: MI = 97.27, RI = 98.15 post-treatment means: MI = 79.92, RI = 95.02. Significant interaction: F (1,72) = 79.630, p < 0.001 Anger-Control: F (1,72) = 22.107, p < 0.001 Pre-treatment means: MI = 24.97, RI = 29.27 post-treatment means: MI = 25.80, RI = 26.35 Anger-Out: F (1,72) = 7.811, p < 0.001 Pre-treatment means: MI = 14.37, RI = 13.22 post-treatment means: MI = 13.95, RI = 14.75Anger-In: Significant reduction across both groups F (1,72) = 4.186, p < 0.05 Pre-treatment mean = 14.83, post-treatment mean = 13.82 |
| (Reingold, 2015) | United States | Pilot study | 42 radiologic technologists | Mindfulness based stress reduction (MBSR) program | Introduction to mindfulness  Mindful eating  Managing anxiety  Physical activity  Journaling  Sustaining mindfulness and planning ahead | 6 week self-administered online program | Immediate post intervention | Not reported | Perceived stress scale (PSS)  American institute of stress (AIs) survey  Occupation stress survey (non-validated) | Changes in stress  Workplace stress perception  Qualitative feedback on stressors and coping strategies | PSS: mean difference = –0.667, P = .02  AIS: mean difference = –2.267, P < .001  Occupational Stress Survey (selected items): mean difference = –0.47, P = .04 |
| (Rice et al., 2024) | United states | Quasi experimental | 20 military personnel | Mindfulness based stress reduction (MBSR) training |  | Delivered either  In person (IP): 8 weekly 2.5 hour sessions and 7 hour silent retreat  Virtual (VW): 8 weekly 1.5 hour sessions and 3.5 hour virtual silent retreat | Immediate post intervention | Not reported | Self-compassion scale (SCS) | Changes in scores of self-compassions | In person: +10% (mean change = 0.33, 95% CI [0.19, 0.47], p < 0.01)  Virtual world: +14% (mean change = 0.35, 95% CI [0.20, 0.51], p < 0.01)  Control group: No significant change (mean change = 0.02, p = 0.76) |
| (Romosiou et al., 2019) | Greece | Quasi experimental | 50 police officers | Integrative psychoeducational group program | Emotional intelligence  Empathy  Resilience  Stress management | Four 4 hour sessions over 5 weeks  Led by trained police officer | Immedate post intervention, 3 month, 2 years | Person centred  Cognitive behavioural psychology approaches | Schutte emotional intelligence scale (SEIS)  Interpersonal reactivity index (IRI)  Connor Davidson resilience scale (CD-RISC)  Perceived stress scale (PSS) | Emotional intelligence  Empathy  Resilience  Perceived stress | Emotional Intelligence (SEIS) Significant Group × Time interaction: F (1.314, 60.443) = 91.443, p < .001, η² = .67  Intervention group improved from M = 135.13 to M = 153.48 post, and M = 151.22 at 3-month follow-up  Control group showed no significant change  Empathy (IRI Subscales)  Perspective Taking: F (1.387, 63.812) = 70.840, p < .001, η² = .61  Fantasy: F (1.439, 66.171) = 23.910, p < .001, η² = .34  Empathic Concern: F (1.349, 62.073) = 16.442, p < .001, η² = .26  Personal Distress: F (1.396, 64.217) = 20.936, p < .001, η² = .31  All empathy subscales improved significantly in the intervention group, no significant change in control group Resilience (CD-RISC) F (1.340, 61.621) = 62.439, p < .001, η² = .58  Perceived Stress (PSS)  F (1.628, 74.883) = 41.948, p < .001, η² = .48 |
| (Rios & Hervas Torres, 2024) | Spain | Pilot study | 9 army personnel | Meaning in life intervention (MLI) | Mindfulness practices  Exploration of personal values strengths and goals  job crafting and meaning in work  visualisation exercises  sharing personal narratives | Six weekly 2 hour face to face group sessions  Facilitated by psychologist | Immediate post intervention | Not reported | Client satisfaction questionnaire (CSQ-8)  Meaning in life questionnaire (MLQ)  Work and meaning inventory (WAMI)  Maslach burnout inventory (MBI)  Utrecht work engagement scale (UWES-9)  Pemberton Happiness Index (PHI)  Patient health questionnaire (PHQ-9) | Acceptability and feasibility  Meaning in life  Meaningful work  Burnout and engagement  Wellbeing  depression | Meaning in Life (Presence): Mean increased from 26.3 to 27.2  Meaning in Life (Search): Mean decreased from 20.4 to 19.2  Meaningful Work: Mean increased from 37.4 to 39.2  Depression (PHQ-9): Mean decreased from 4.6 to 3.9  Well-being (PHI): Mean increased from 8.4 to 8.7 |
| (Rosenbaum et al., 2022) | Australia | Nonrandomised trial | 60 police officers | RECONNECT exercise program | Not reported | 12 week individualised exercise program, conducted twice weekly  Delivered by physiotherapists | Baseline, week 6, week 12 | Not reported | PTSD checklist (PCL-5)  Depression, anxiety and stress scale (DASS-21)  Insomnia severity index (ISI)  Alcohol use disorders (AUDIT) | PTSD symptoms  Depression  Anxiety  Stress  Insomnia  Alcohol use | PTSD (PCL-5) F (2, 59.96) = 12.93, p < .001Depression (DASS) F (2, 73.39) = 4.42, p < .05 d = 0.71  Anxiety (DASS)F (2, 61.98) = 5.70, p < .01  d = 0.55 Stress (DASS) F (2, 72.478) = 5.44, p < .01 d = 0.69 Insomnia (ISI)F (2, 69.68) = 3.00, p = .056 d = 0.50Alcohol Use (AUDIT) No significant change F (2, 27.92) = 0.09, p > .05 |
| (Said et al., 2022) | Palestine | Nonrandomised trial | 150 nurses | Modified RAPID-PFA (psychological first aid) training program | Interactive lectures  Group discussions  Scenario based simulations and role playing  Emphasis on connectedness social support and faith | 9 hours delivered over 5 weeks  Delivered by licensed psychologist | Immediate post intervention | John Hopkins RAPID PFA model | Psychological preparedness for disaster threat scale (PPDTS)  General self-efficacy scale (GSE)  Self-esteem scale (SES)  Life orientation test (LOT)  State trait anxiety inventory  PTSD diagnostic scale (PDS-5)  PFA evaluation | Psychological preparedness (PPDTS)  Self-efficacy  Self esteem  Optimism  Trait anxiety  PTSD  PFA related knowledge skills and attitudes | Psychological Preparedness (PPDTS) Significant group-by-time effect: B = 4.9, 95% CI [1.16, 9.74], p = 0.013  Mean post-test scores: Intervention = 37.81, Control = 32.64 Effect size: Cohen’s d = 1.41 (large) Optimism (LOT) Group-by-time effect: B = 0.16, 95% CI [−1.76, 2.08], p = 0.009 Self-Efficacy (GSE) Group-by-time effect: B = 2.3, 95% CI [0.18, 4.41], p = 0.033 Self-Esteem (SES) Time effect: B = 0.23, 95% CI [−0.21, 0.67], p = 0.008 Trait Anxiety Group-by-time effect: B = −7.33, 95% CI [−10.66, −3.99], p < 0.001 PTSD No significant group-by-time effect: B = 1.53, 95% CI [−4.07, 7.12], p = 0.593 PFA Knowledge, Skills, Attitudes Knowledge: B = 4.68, 95% CI [3.63, 5.73], p < 0.001  Skills: B = 6.86, 95% CI [2.14, 11.57], p = 0.004  Attitudes: B = 6.65, 95% CI [2.31, 11], p = 0.003 |
| (Scotland-Coogan et al., 2020) | United States | Nonrandomised trial | 71 army personnel | Dog training program | Veterans train their own service dogs  Training included obedience commands, public behaviour and canine good citizen requirement  Included supportive counselling from clinical social worker  Included psychoeducational support and peer interaction | Weekly 60 minute sessions of 10 participants per group  Led by certified master dog trainer | Immediate post intervention | Not reported | Trauma symptom inventory (TSI-2) | PTSD symptoms and trauma related psychological problems | Self-disturbance: M change = –21.83, t (53) = 8.35  Posttraumatic stress: M change = –24.61, t (52) = 8.42  Externalisation: M change = –22.46, t (47) = 6.73  Somatisation: M change = –4.36, t (53) = 5.57  Clinical Scales  Anxious arousal: M change = –6.56, t (54) = 8.03  Depression: M change = –8.05, t (54) = 8.66  Anger: M change = –6.90, t (52) = 8.10  Intrusive experiences: M change = –6.49, t (53) = 7.74  Defensive avoidance: M change = –5.39, t (53) = 7.22  Dissociation: M change = –6.51, t (54) = 8.07  Suicidality: M change = –4.13, t (54) = 4.54  Insecure attachment: M change = –7.34, t (53) = 7.32  Impaired self-reference: M change = –6.97, t (54) = 8.18  Tension reduction behaviour: M change = –6.72, t (52) = 6.75 |
| (Sloan et al., 2022) | United States | Randomised control trial | 19 army personnel | Written exposure therapy (WET) vs Cognitive processing therapy (CPT) | WET: involves writing about trauma  CPT: cognitive restructuring | WET: 5 weekly sessions.  CPT: 12 sessions  All sessions in person and delivered by therapist | Baseline, 10, 20 and 30 weeks | Not reported | PTSD scale (CAPS-5) | PTSD symptoms | PTSD Symptom Reduction (CAPS-5) WET was noninferior to CPT at all time points (10, 20, 30 weeks)  Mean difference in symptom reduction (WET vs CPT):  Week 30: 0.33 (SE = 2.58), upper limit = 4.59  Effect Sizes (Cohen’s d)  Within-group:  WET: 0.48–0.54 (medium)  CPT: 0.78–0.95 (medium to large)  Between-group: 0.01–0.22 Reliable Change (≥12-point reduction on CAPS-5 at 30 weeks) WET: 47.2% (17/36)  CPT: 37.5% (12/32)  Not statistically significant (χ² = 0.66, p = .42) |
| (Smeeding et al., 2010) | United States | Longitudinal study | 165 army personnel | Integrative Health Clinic and Program (IHCP)  Complementary and alternative medicine therapies and mind body skills classes  Includes acupuncture, aquatic body work, stress management, hypnosis, meditation, yoga. | Biopsychosocial program | 10 sessions  Therapies delivered by credentialled professionals | 6 months, 1 year, 2 years | Not reported | Beck Depression inventory (BDI)  Beck Anxiety inventory (BAI)  Health Related quality of life (HRQOL)  Numerical rating scale for stress (NRS) | Depression  Anxiety  Health related quality of life | Anxiety: Cohen’s d = 0.41 at 6 months (95% CI: –7.5 to –2.6)  Depression: d = 0.31 at 6 months (95% CI: –6.1 to –0.9)  HRQOL (Health Transition): d = 0.43 at 6 months  Between-group difference in Bodily Pain: p = 0.043, d = 0.32  HRQOL improvements in Role Emotional (d = 0.32), Vitality (d = 0.27), Bodily Pain (d = 0.25), Health Transition (d = 0.52) |
| (Stelnicki et al., 2021) | Canada | Program evaluation | 136 public safety personnel  Firefighters 33  Paramedics 25  Police officers 15  Crown prosecutors 11 | Before operational stress program (BOS) | Psychoeducational program  Focused on self-awareness, emotional regulation, communication and functional disconnection/reconnection | 8 week group based programme followed by 10 monthly maintenance sessions  Group sessions up to 12 participants  Delivered in person and virtually | Baseline, post gram, 1,4,7 and 1o months | Cognitive behaviour therapy (Beck, 1976; Beck et al., 1985) | PTSD checklist (PCL-5)  Depression anxiety stress scale (DASS-21)  Alcohol use disorders identification test (AUDIT)  Difficulties in emotional regulation scale (DERS)  Worl health organisation quality of life (WHOWOL)  Social provisions scale (SPS-10)  Opening minds survey for workplace attitudes (OMS-WA)  Guilt and shame proneness scale (GASP)  Brief resilience scale (BRS) | PTSD  Depression  Anxiety  Stress  Alcohol use  Emotion regulation  Guilt  Shame  Stigma  Social support  Quality of life  Resilience | Statistically Significant Improvements (Time 1 to Time 4) PTSD: γ = −1.84, p < 0.05, ES = 0.11  Quality of Life: γ = 1.11, p < 0.05, ES = 0.09  Social Support: γ = 0.78, p < 0.05, ES = 0.17  Stigma: γ = −1.01, p < 0.01, ES = 0.18 |
| (Stetz et al., 2011) | United States | Randomised control trial | 60 army personnel | Technology assisted relaxation training using virtual reality (VR) | Three 7 minute VR video clips embedded audio guiding progression muscle relaxation and controlled breathing | Daily morning in person VR sessions for 3 days  Evening self-practice using portable video players  Instructions and logs for self-practice | Pre and post intervention assessment | Not reported | PTSD checklist (PCL-M)  State trait anxiety inventory (STAI-STATE)  Universite du Quebec Outaouais presence questionnaire (UQO-PQ) | Change in anxiety  Anxiety response during simulated stressful tasks | STAI-State: VR group: Mean = 2.19, SD = 0.21 Control: Mean = 2.34, SD = 0.21 ANCOVA: F (1,40) = 5.77, p = 0.021, partial η² = 0.13 (significant reduction in EG)  Stressful task comparison (pre vs. post): No significant difference ANCOVA: F (1,48) = 0.04, p = 0.84, partial η² = 0.001 |
| (Stoller et al., 2012) | United States | Randomised control trial | 70 army personnel | Sensory enhanced hatha yoga | Focused on breathing, poses, meditation, relaxation.  Enhanced sensory input  Use of straps and blocks and calming music  Positive affirmations and readings | 3 week program with 75 minute sessions, 7 days a week  Conducted in a gym in Iraq  Led by certified yoga instructor | Pretest and post-test assessment | Not reported | Adolescent sensory profile (AASP)  State trait anxiety inventory (STAI)  Quality of life survey | Sensory processing (AASP)  State and trait anxiety (STAI)  Quality of life indicators | Anxiety (STAI) Difference = 9.61, t (67) = 4.68, p < .001 Anxiety: Group Difference = 8.06, t (67) = 4.73, p < .001 Sensory Processing (AASP) No significant differenceQuality of Life Survey (18 items) Treatment group showed significantly greater improvement on 16 of 18 items |
| (Turan & Canbulat, 2023) | Turkey | Randomised control trial | 57 nurses | Training program on accepting and expressing emotions | Topics included emotional awareness, emotion thought behaviour links, body language, empathy and emotion acceptance | 9 week training program in person  Weekly 75 minute group sessions  Delivered by trained psychiatric nurse | Pre training  Post training  6 months  12 months  24 months | Cognitive behavioural therapy | Resilience scale for adults (RSA)  Beck depression inventory (BDI) | Psychological resilience (RSA)  Depression levels (BDI) | Psychological Resilience (RSA) Significant increase Group × time interaction: p < 0.001  No significant change in control group over time (p > 0.05)  Depression (BDI)  Significant decrease Group × time interaction: p < 0.001  No significant change in control group over time (p > 0.05) |
| (Van Der Meer et al., 2020) | Netherlands | Randomised control trial | 287 health care professionals | SUPPORT coach: a Dutch adaptation of the US PTSD coach mobile app | Self-help mobile app with five sections  Psychoeducation on trauma and PTSS  Support recourses  Self-assessment  Calendar for scheduling activities  CBT based symptom management tools | One month unlimited access to the app without guidance  Participants used their own smart phone or devices | Baseline (T1)  Post intervention 1 month (T2)  2 months (T3) | Cognitive behavioural therapy | PTSD checklist (PCL-5)  Post traumatic cognitions inventory (PTCI)  Resilience evaluation scale (RES)  Social support list (SSL-6) | Primary change in PTSD  Changed in negative trauma related cognitions  Psychological resilience  Social support | PTSD Symptoms No significant difference T2–T1: U = 4497.50, p = .44, r = –.05. T3–T1: U = 3714.50, p = .31, r = –.07 Negative Trauma-Related Cognitions (PTCI) Significant reduction T2–T1: U = 3681.50, p = .01, r = –.18. T3–T1: U = 3081.50, p = .006, r = –.20 Psychological Resilience Significant greater increase in intervention group at T3:  T3–T1: U = 3407.50, p = .047, r = –.15  Social Support  No significant difference T2–T1: U = 4358.00, p = .76, r = –.02. T3–T1: U = 3768.00, p = .66, r = –.04 |
| (Walker et al., 2024) | United States | Randomised control trial | 161 army personnel | Motivational Enhancement Therapy (MET) | Sessions used motivational interviewing techniques  Sessions focused on increasing motivation, addressing barriers, and facilitating treatment engagement | 1-2 telephone sessions (30-090 minutes)  Delivered via telephone by trained counsellors | Baseline  6 weeks  3 months  6 months | Not reported | Clinician administered PTSD scale CAPS-5  Treatment reactions scale (TRS)  Readiness Ruler  Custom treatment uptake questionnaire | PTSD treatment uptake  PTSD symptom severity  PTSD diagnosis  Treatment stigma  Perceived treatment efficacy  Readiness to change | PTSD Symptom Severity (CAPS-5) Both groups showed significant reductions over time:  MET: Baseline M = 33.4. 6-month M = 18.3  TAU: Baseline M = 32.4. 6-month M = 20.8  No overall significant difference between MET and TAU PTSD Diagnosis (CAPS-5) At 6 months:  MET: 29.7% met PTSD criteria  TAU: 40.5% met PTSD criteria  Difference not statistically significant Treatment Reactions (Stigma and Perceptions) No significant differences between MET and TAU |
| (Walter et al., 2023) | United States | Randomised control trial | 96 army personnel | Surf therapy and Hike therapy | Group based outdoor physical activity in natural environments  Surf therapy at a public beach, Hike therapy at various San Diego County locations | 6 week programs with weekly 3-4 hour sessions  Delivered with trained facilitators | Baseline  2 weeks post intervention  3 months | Not reported | Generalised anxiety disorder scale (GAD7)  Positive and negative affect schedule (PANAS)  Response to stressful events scale (RSES4)  Numerical pain rating scale (NPRS)  Short Form Health Survey (SF-36v2)  Structured diagnostic interview for MDD and PTSD (MINI7)  PTSD checklist (PCL5)  International physical activity questionnaire (IPAQSF) | Anxiety  Positive affect  Negative affect  Resilience  Pain  Physical functioning  Social functioning | Anxiety (GAD-7) MD = –3.07, p < 0.001 No significant difference between Surf and Hike Therapy: p = 0.387  Positive Affect (PANAS - PAS)  No significant change from pre- to post program: p = 0.053 Negative Affect (PANAS - NAS) Significant reduction from pre- to post program: MD = –3.66, p < 0.001  No significant difference between interventions: p = 0.548  Resilience (RSES-4)  Significant increase from pre- to post program: MD = +0.98, p = 0.013  No significant difference between interventions: p = 0.136 Pain (NPRS) No significant change over program duration: p = 0.776  Significant within-session change: MD = +0.23, p = 0.036  Surf Therapy showed less increase in pain than Hike Therapy: Time × Intervention, p < 0.001 Physical Functioning (SF-36 PF) No significant change: p = 0.055 Social Functioning (SF-36 SF) Significant improvement from pre- to post program: MD = +5.86, p < 0.001  No significant difference between interventions: p = 0.208 |
| (Walter et al., 2019) | United states | Longitudinal study | 74 army personnel | Surf therapy program | No formal psychotherapy component, experiential outdoor activity model | 6 week program with weekly 3-4 hour group surf sessions at the beach  Delivered with trained volunteer surf instructors | Baseline  6 weeks post intervention  Before and after each weekly session (14 assessments) | Not reported | Patient health questionnaire (PHQ-8)  Generalised anxiety disorder (GAD7)  PTSD checklist (PCL5)  Life events checklist (LEC5)  Positive and negative affect schedule (PANAS)  Pain rating scale (NPRS)  Insomnia severity index (ISI)  Client satisfaction questionnaire (CSQ8) | Depression  Anxiety  PTSD symptoms  Positive and negative affect  Pain  Insomnia  Depression  Satisfaction with intervention | Depression (PHQ-8) β = –2.31, p < .01, d = 0.42Anxiety (GAD-7) β = –3.55, p < .001, d = 0.61Positive Affect (PANAS - PAS) β = +9.46, p < .001, d = 0.78Negative Affect (PANAS - NAS) β = –6.40, p < .001, d = 0.60Pain (NPRS) and Insomnia (ISI) No significant changes pre- to post-program or within sessions Program Satisfaction (CSQ-8) Mean score: 31.0 out of 32 (SD = 1.6) |
| (Wang et al., 2024) | China | Feasibility randomised controlled trial | 96 healthcare workers | READ-Y PFA | Culturally adapted Psychological First Aid (PFA) training program  Included five components: Rapport, evaluation, aid, disposition and self-care | 1 day in person training (5 sessions of 75 minutes)  4 online group supervision sessions over 3 months  Delivered by trained mental health professionals using blended learning and simulation | Baseline (T0)  2 weeks (T1)  2 months (T2)  3 months (T3)  Post training | Not reported | Knowledge skills and attitude survey (KSA)  Depression, anxiety stress scale (DASS-21)  Brief resilience scale (BRS)  General self-efficacy scale (GSE)  Simplified coping style questionnaire (SCSQ)  Professional quality of life (ProQOL)  Post traumatic growth inventory (PTGI) | recruitment  retention  adherence  Satisfaction on usage  Depression  Anxiety  Stress  Burnout  Resilience  Self-efficacy  Coping  Compassion satisfaction  Post traumatic growth | Depression: F (2,232) = 2.874, p = .046, η² = .031  Burnout: F (2,211) = 3.729, p = .018, η² = .037  KSA: F (1,137) = 6.378, p = .038, η² = .075 |
| (Watson & Andrews, 2018) | United Kingdom | Cross sectional | 859 police officers | Trauma Risk Management (TRiM) | A peer support system  Includes follow up and referral to mental health professionals if needed | Delivered by trained peer practitioner within the police force  Trim had been used for 7-12 years in participating forces | N/A | Not reported | Online survey  PTSD checklist (PCL-C)  Stigma and barriers to care questionnaire  Military Stigma Scale | PTSD  Barriers to seeking help  Self-stigma  Public Stigma | Lower PTSD symptoms: Median PCL-C score: TRiM = 23, non-TRiM = 26. Mann–Whitney U = 41,363.50, p < .001  Fewer barriers to help-seeking: TRiM mean = 25.58 (SD = 5.88), non-TRiM mean = 28.19 (SD = 6.39) t (857) = 5.04, p < .001  Lower public stigma: TRiM mean = 37.70 (SD = 9.96), non-TRiM mean = 40.92 (SD = 10.26) t (857) = 3.72, p < .001 |
| (Wesemann et al., 2016) | Germany | Randomised controlled trial | 67 army personnel | CHARLY (Chaos Driven Situations Management Retrieval System) | 1.5 day computer based, biofeedback enhanced training programme  Included psychoeducation, stress management, coping strategies and serious gaming  Uses photo realistic simulations (Not VR) and biofeedback (skin conductance) | CHARLY group vs control group who received conventional psychoeducational training by a psychologist | T1 before training  T2 after training  T3 4-6 weeks post deployment | preventive learning processes | Brief symptom inventory (BSI)  Attitude questionnaire (ESG)  PTSD and deployment stress (ESG Developed)  Combat experiences scale (MHAT)  Post Traumatic stress diagnostic scale (PDS) | Attitudes toward PTSD  Knowledge of PTSD  General mental state  PTSD symptoms  Exposure to critical incidents | **Attitude Change**: F (2,46) = 3.33, p = 0.045, η² = 0.126  **Knowledge Gain**: F (2,50) = 9.757, p < 0.001, η² = 0.281  **Mental State (BSI-GSI)**: F (2,49) = 3.654, p = 0.033, η² = 0.130  **PTSD Symptoms (PDS at t3 only)**:  Re-experiencing: t (36) = −2.196, p = 0.020, ω² = 0.091  Avoidance: t (34) = −1.860, p = 0.039, ω² = 0.064  Arousal: t (34) = −1.971, p = 0.031, ω² = 0.074  Global impairment: t (34) = −2.032, p = 0.028, ω² = 0.08  **Critical Incidents (MHAT)**:  No significant group difference post-deployment:  F (1,51) = 2.71, p = 0.11 |
| (Wild et al., 2020) | United Kingdom | Randomised control trial | 430 emergency responders:  225 police  120 paramedics  68 firefighters  17 Search and rescue | Group based resilience intervention | Content included stress management, mindfulness, relaxation techniques and psychoeducation | Six weekly group 2.5 hour sessions  Delivered by trained facilitators with weekly supervision  Coparison with psychoeducation | Baseline  6 weeks post intervention  3 month follow up | model of resilience | Warwick Edinburgh Mental wellbeing scale (WEMWBS)  Connor Davidson Resilience Scale (CD-RISC)  Genera self-efficacy scale (GSE)  Social participation scale  Social support scales  Confidence in managing mental health  Days off work due to illness  Depressive attributions questionnaire  Brief coping behaviour questionnaire  Responses to intrusions questionnaire  Ruminative response scale  PTSD symptoms (PCL-5)  Depression (PHQ-9)  Anxiety (GAD-7)  AUDIT (alcohol use)  Problem solving questionnaire  Eysenck personality questionnaire | Wellbeing  Resilience  Self-efficacy  Social capital  Confidence in managing mental health  Days off work  Coping strategies  Rumination  PTSD  Depression  Anxiety  Alcohol use | **No significant differences**  Wellbeing (WEMWBS) adjusted group difference at post = −0.27 (SE = 0.85), p = .755  Resilience (CD-RISC) adjusted group difference at post = 0.52 (SE = 1.41), p = .712  Days off work: Mann-Whitney U = 11,684.00, p = .892 (post); U = 13,715.00, p = .754 (follow-up) |
| (Wu et al., 2012) | China | Randomised control trial | 1267 army personnel | 512 psychological model (512 PIM) | Five stage group intervention  Introduction, facts and thoughts, reaction and symptoms, stress management, cohesion training.  Compared with critical incident stress debriefing and no intervention | 2 hour five stage group intervention  Delivered by trained clinical psychologists  Conducted 1 month post trauma | Baseline  1 month  2 months  4 months post intervention | Not reported | Structured interview for PTSD (SI PTSD)  Hospital Anxiety and depression scale (HADS) | PTSD  Anxiety  Depression | PTSD (SI-PTSD total scores):  4 months: F = 28.77, df = 1,127, p < 0.01  Estimated reduction (1 to 4 months):  512 PIM: 14.1 (95% CI: 9.3–18.9)  Debriefing: 10.9 (95% CI: 6.3–15.5)  Control: 10.2 (95% CI: 6.1–14.3)  Anxiety (HADS-A):  Main effect of group: F = 3.11, df = 1,141, p < 0.01  Reduction (1 to 4 months):  512 PIM: 2.5 (95% CI: 1.5–3.2)  Debriefing: 1.6 (95% CI: 0.7–2.6)  Control: 1.7 (95% CI: 0.7–2.5)  Depression (HADS-D):  Main effect of group: F = 2.06, df = 1,141, p < 0.01  Reduction (1 to 4 months):  512 PIM: 2.3 (95% CI: 1.3–3.3)  Debriefing: 1.4 (95% CI: 0.5–2.3)  Control: 1.8 (95% CI: 0.8–2.8)  PTSD Diagnosis Rates:  Baseline: 8.4%  1 month: 4.6%  2 months: 3.3%  4 months: 2.6%  No significant differences in diagnosis rates between groups. |
| (Young-McCaughan et al., 2022) | United States | Randomised control trial | 72 army personnel | Four conditions:  Exercise only  Imaginal exposure  Imaginal exposure and exercise  Self-care (control)   - - 1. E | Exercise: 20–25 minutes, 5×/week, at >60% heart rate reserve  Imaginal exposure: 5 weekly sessions of trauma-focused exposure and emotional processing  Combined: Imaginal exposure audio played during aerobic exercise  Control: Nurse-led self-care | 5 sessions over 8 weeks  1 hour each  Delivered by trained therapists, exercise specialists or nurses | Baseline  Post treatment  1 month follow up  6 month follow up | Not reported | Stressor specific version (PCL-S)  PTSD symptom scale interview (PSS-I)  Beck Depression inventory (BDI-II)  Beck Anxiety Inventory (BAI)  State trait anger expression inventory (STAXI2)  Alcohol use disorders identification test (AUDIT)  Scale for suicidal ideation (SSI) | PTSD symptom severity  Depression  Anxiety  Anger  Alcohol use  Suicidality | PTSD symptoms (PCL-S):  Main effect of time: F = 14.69, p < .0001  No significant differences between groups (F = 0.43, p = .73)  All groups showed significant reductions from baseline:  Exercise-only: d = 0.71  Imaginal-only: d = 0.73  Imaginal + exercise: d = 0.74  Control: d = 0.45  Mean PCL-S reduction:  Exercise-only: 47.0 → 36.2  Imaginal-only: 45.5 → 34.4  Imaginal + exercise: 45.7 → 34.3  Control: 47.6 → 40.7  Clinically significant reduction (≥10 points on PCL-S):  Post-treatment: 49%  1-month: 48%  6-month: 65%  No significant differences between groups in:  Depression (BDI-II)  Anxiety (BAI)  Anger (STAXI-2) |
| (Zarvijani et al., 2021) | Iran | Randomised control trial | 70 nurses | Acceptance and commitment therapy (ACT) | Focused on increasing psychological flexibility and reducing stress | 8 sessions 2 hours each  Delivered by ACT therapist | Pretest  1 month after intervention | Steven Hayes Acceptance commitment therapy model | Perceived stress scale (PSS-14)  Acceptance and action questionnaire (AAQII) | Perceived stress (PS)  Psychological flexibility (PF) | Pre-test scores (Mean ± SD):  PS: Experimental = 23.42 ± 6.02; Control = 23.36 ± 6.38  PF: Experimental = 47.13 ± 9.42; Control = 47.63 ± 7.56  Post-test scores (Mean ± SD):  PS: Experimental = 18.66 ± 5.44; Control = 23.20 ± 5.70  PF: Experimental = 55.49 ± 9.55; Control = 47.56 ± 9.42  ANCOVA results:  PF post-test: F = 37.8, p < 0.001  PS post-test: F = 28.36, p < 0.001 |

| Author | Country | Study Type | Population | Intervention | Description of Intervention | Data collection tool | Outcomes | Results |
| --- | --- | --- | --- | --- | --- | --- | --- | --- |
| (Carlier et al., 2000) | Netherlands | Control Group Design | 243 police officers | Critical Incident Stress Debriefing (CISD) | Three debriefing sessions held at 24 hours, 1 month, and 4 months.  Included traumatic stress education and followed a seven stage, semi structured procedure. | The specific tool or scale used for self-reporting was not detailed | Levels of satisfaction | **First and Second Sessions**: 98% of respondents were satisfied, and 2% were satisfied to a degree.  **Third Session**: 88% were satisfied, and 12% were satisfied to a degree. |
| (Eddy et al., 2021) | USA | Qualitative | 5 police officers | Mindfulness Based Resilience Training (MBRT) | 8 week program, 2 hour sessions. It includes experiential and didactic exercises such as body scan, sitting and walking meditation, mindful movement and group discussions. Content and language adapted specifically for law enforcement.  Delivered by certified mindfulness trainer and police lieutenant. | Semi structured interviews conducted post MBRT | Perceived improvements in intrapersonal and interpersonal functioning, benefits of MBRT, strategies for overcoming potential barriers of mindfulness practice. | **Intrapersonal Functioning**: Participants reported increased relaxation, awareness of the present moment, and improved physical well-being.  **Interpersonal Functioning**: Participants noted increased nonreactivity, improved family communication, and enhanced camaraderie with colleagues.  **Most Helpful Elements**: Dedicated practice space, body scan, mindful movement, and informal mindfulness practices.  **Least Helpful Elements**: Walking meditation was found to be less helpful and challenging.  **Obstacles**: Scheduling conflicts and cultural stigma were identified as barriers.  **Overcoming Obstacles**: Encouragement from facilitators and adaptations to practice settings were helpful.  **Suggested Improvements**: Participants expressed interest in ongoing trainings or booster sessions. |
| (Janes et al., 2022) | United Kingdom | Qualitative | 23 health professionals  (midwives, paediatricians, obstetricians, gynaecologists, paramedics, physician associates, mammographers, sonographers) | Dual element resilience based intervention | 3.5 hour interactive group workshop and 1 follow-up one hour coaching phone call. Workshop included work based case studies. | Interviews | Perceptions of psychological resilience, relevance of the intervention, impact on ability to cope with error | **Shifting Perspectives on Resilience**: Participants reported mixed and complex feelings about resilience, noting that it was often misunderstood and perceived negatively due to previous training experiences. The intervention helped shift their understanding, emphasising the importance of addressing both individual and system-level factors.  **Humanising Clinical Work**: The intervention helped normalise and legitimize the emotional burden of clinical work, making participants feel less isolated in their experiences.  **Resilience as Pervasive Across Personal and Professional Life**: Participants found the skills learned in the intervention applicable to both their professional and personal lives, creating a "virtuous circle" of resilience.  **Resilience Building as Personal Development**: The intervention was seen as valuable for personal development, though participants noted that readiness to engage with the topic varied.  **Resilience as Contextual and Multi-Layered**: Resilience was viewed as a complex concept influenced by individual, organizational, and system-level factors. |
| (Korpela & Nordquist, 2024) | Finland | Qualitative | 15 Firefighters/ paramedics | Post Critical Incident Seminar (PCIS) | 3 day residential seminar  Included psychoeducation, peer support, and two individual sessions with a psychotherapist (with optional EMDR)  Delivered by multidisciplinary team: seminar director, psychotherapists, peer support members, a priest and a trainee | Interviews | Participants experience of the impact of PCIS on their critical incident related experiences and psychological state | **Social Changes**  Improved openness and communication with family and peers  Increased willingness to seek and offer help  Enhanced peer support and shared experiences  **New Perspectives and Sensations**  Improved well-being and self-awareness  Reduced anxiety, fear, and physical symptoms  Greater compassion and acceptance toward self and others  **Incident-Related Components**  Normalisation of reactions to trauma  Reduced emotional intensity and burden of memories  Acceptance and integration of the incident into personal narrative  **Future-Oriented Processes**  Life changes (e.g., career shifts, taking leave)  Ongoing recovery and hope for the future  Awareness of continued need for processing  **New Abilities and Actions**  Enhanced emotional regulation and empathy  Increased professional confidence and resilience  Adoption of self-care practices and boundary setting |
| (Leonard & Alison, 1999) | Australia | Nonrandomised trial with qualitative element | 20 police officers | Critical Incident Stress Debriefing (CISD) | Conducted within 72 hours post shooting incident  One time group session facilitated by trained professionals | Survey | Satisfaction with support | Mixed views on CISD effectiveness  Common themes: lack of follow-up, poor communication, increased awareness of emotional responses |
| (Lynch et al., 2018) | Ireland | Qualitative | 10 emergency department staff. Doctors, nurses, allied health professionals, administrative staff | Mantra Meditation (MM) programme | Four sessions, lasting 4 hours each. Delivered over 6 week period  Conducted in the hospital, facilitated by a healthcare expert and meditation expert | Interview | Perceived impact of MM programme  Perception of Emergency working environment  Barriers and facilitators to meditation practice | **Work Pressure and Perceived Stress**  High levels of burnout, exhaustion, and emotional strain  Need for workplace well-being interventions  **Perceived Benefits of Meditation**  Increased attention and awareness: “It just kind of settles you and kind of focuses you…”  Improved emotion regulation and coping: “I think my coping mechanisms at work are a lot better.”  Better sleep and relaxation: “I feel way better in myself. My sleep has improved.”  **Conflicting Attitudes to Practice**  Feelings of guilt for taking time to meditate  Difficulty prioritising self-care  **Barriers to Practice**  Shift work and unpredictable schedules  Length of practice (20 minutes twice daily seen as too long)  Personality traits (e.g., “go, go, go” types found it hard to sit still)  **Facilitators to Practice**  Organisational support (e.g., protected time and space)  Interpersonal support (e.g., group meditation, peer encouragement) |
| (McLean, Miller, et al., 2024) | United States | Mixed methods | 29 military personal | Web Based Prolonged Exposure Therapy (web-PE) | 10 sessions, self-guided, therapist facilitated online program  Included breathing retraining, psychoeducation, in vivo and imaginal exposure and homework review | Telephone Interview | Experience of using Wen-PE | **Helpfulness**  Most participants found Web-PE helpful in reducing PTSD symptoms.  Participants reported gaining insight and being able to process traumatic memories.  **Advantages of Online Therapy**  Participants appreciated the flexibility of completing sessions at home and on their own schedule.  Some felt safer and more open during therapy due to the privacy and anonymity of the online format.  **Self-Accountability (72%)**  Barriers included busy schedules, work and family demands, and difficulty staying motivated. **Suggested Improvements** Participants recommended:  Adding more video content and relatable case examples.  Increasing therapist support or adding group sessions.  Extending the program beyond 10 sessions. **Sceptical/Difficult at First** Many were initially sceptical about the effectiveness of online therapy or found the exposure exercises difficult.  However, most reported that their perceptions improved as they progressed through the program. **Technical Issues**  Participants reported glitches such as freezing, slow performance, and login issues.  The severity and impact of these issues varied across individuals. **Valued Therapist Support**  Therapist support was highly appreciated.  Participants described therapists as knowledgeable, responsive, and encouraging.  Some participants desired more frequent or deeper therapist interaction |
| (Stelnicki et al., 2021) | United states | Process evaluation | 136 public safety personnel  Firefighters 33  Paramedics 25  Police officers 15  Crown prosecutors 11 | Before operational stress program (BOS) | 8 week group based programme followed by 10 monthly maintenance sessions  Group sessions up to 12 participants  Delivered in person and virtually | Qualitative survey | Experience of using BOS | Participants reported improvements in:  Self-awareness and emotional insight  Coping and behavioural changes  Relationships with family and colleagues  Feeling less alone and more supported  Some participants reported temporary symptom worsening due to increased awareness |
| (Stetz et al., 2011) | United States | Randomised control trial | 60 army personnel | Technology assisted relaxation training using virtual reality (VR) | Three 7 minute VR video clips embedded audio guiding progression muscle relaxation and controlled breathing | Focus group  Questionnaire | Experience using VR intervention | 79% of EG participants reported feeling relaxed or calm  53% preferred immersive VR training  50% intended to continue using the techniques  Suggestions included longer sessions and more flexible scheduling |
