# Supplementary material for: Mental health and wellbeing interventions for uniformed service personnel: a mixed methods systematic review
Source: BMC Med. 2026 Apr 17;24:333. doi: 10.1186/s12916-026-04811-1 (PMC13217766; doi:10.1186/s12916-026-04811-1)
Supplement: Supplementary file 4 — Additional file 4: Table 4 Risk of bias assessments. [file 12916_2026_4811_MOESM4_ESM.docx]

| **Author** | **Risk of bias assessment** |
| --- | --- |
| (Abbasalizadeh et al., 2024) | Low |
| (Adler et al., 2009) | Low |
| (Alghamdi et al., 2015) | Medium |
| (Arnetz et al., 2009) | Low |
| (Blevins et al., 2011) | Medium |
| (Brown et al., 2019) | Medium |
| (Carlier et al., 2000) | Low |
| (Chitra & Karunanidhi, 2021) | Low |
| (Christopher et al., 2018) | Medium |
| (Chu et al., 2022) | High |
| (Villaruz Fisak et al., 2020) | High |
| (Foa et al., 2018) | Medium |
| (Foa et al., 2022) | Medium |
| (Frappell-Cooke et al., 2010) | High |
| (Garner, 2008) | Medium |
| (Gerdes et al., 2022) | Medium |
| (Giaume et al., 2024) | Medium |
| (Gon et al., 2023) | Low |
| (Grupe et al., 2021) | Low |
| (Hsu et al., 2021) | Low |
| (Jones et al., 2021) | Medium |
| (Jones et al., 2013) | Medium |
| (Joyce et al., 2019) | Medium |
| (Judkins & Bradley, 2017) | Medium |
| (Khatib et al., 2022) | Medium |
| (Kline et al., 2024) | Medium |
| (Krick & Felfe, 2020) | Medium |
| (Krick & Felfe, 2024) | Medium |
| (Leggett et al., 2013) | Medium |
| (Leonard & Alison, 1999) | Medium |
| (Mackintosh et al., 2017) | Low |
| (Maguire et al., 2024) | Medium |
| (Mainsbridge et al., 2020) | Low |
| (Maloney et al., 2024) | Medium |
| (Markwell et al., 2016) | High |
| (McCall, 2023) | High |
| (McKeon et al., 2023) | Medium |
| (McLean, Cook, et al., 2024) | Low |
| (Meland et al., 2015) | Medium |
| (Millegan et al., 2021) | Medium |
| (Mohr et al., 2024) | Low |
| (Morland et al., 2016) | Medium |
| (Márquez et al., 2021) | Low |
| (Narayanan et al., 2024) | Medium |
| (Nassif et al., 2023) | Low |
| (Navarrete et al., 2022) | Medium |
| (Niemeyer et al., 2020) | High |
| (Nimenko & Simpson, 2014) | Medium |
| (Nwokeoma et al., 2019) | Low |
| (Onyishi et al., 2021) | Medium |
| (Otis et al., 2024) | Medium |
| (Pallavicini et al., 2022) | Medium |
| (Peng et al., 2024) | Medium |
| (Peterson et al., 2023) | Low |
| (Price et al., 2022) | Medium |
| (Rajeswari et al., 2020) | High |
| (Ramey et al., 2016) | High |
| (Ranta, 2012) | High |
| (Reingold, 2015) | High |
| (Rice et al., 2024) | Medium |
| (Romosiou et al., 2019) | Medium |
| (Rios & Hervas Torres, 2024) | Medium |
| (Rosenbaum et al., 2022) | High |
| (Said et al., 2022) | Medium |
| (Scotland-Coogan et al., 2020) | High |
| (Sloan et al., 2022) | Medium |
| (Smeeding et al., 2010) | Medium |
| (Stelnicki et al., 2021) | Medium |
| (Stetz et al., 2011) | High |
| (Stoller et al., 2012) | High |
| (Turan & Canbulat, 2023) | High |
| (Van Der Meer et al., 2020) | High |
| (Walker et al., 2024) | Medium |
| (Walter et al., 2023) | Low |
| (Walter et al., 2019) | High |
| (Wang et al., 2024) | Low |
| (Watson & Andrews, 2018) | Low |
| (Wesemann et al., 2016) | Medium |
| (Wild et al., 2020) | Medium |
| (Wu et al., 2012) | Low |
| (Young-McCaughan et al., 2022) | Low |
| (Zarvijani et al., 2021) | Low |

# **Key**

| Low |
| --- |
| Medium |
| High |
